# Trimeric hemagglutinin vaccine provides chickens complete protection against lethal H5 subtype avian influenza virus from clade 2.3.4.4b

**DOI:** 10.1080/22221751.2026.2678649

**Published:** 2026-06-10

**Authors:** Taoran Chen, Qiao Huang, Xingtao Chen, Jiahang Zhu, Deming Kong, Xiuying Liao, Ming Liao, Huiying Fan

**Affiliations:** a College of Veterinary Medicine, South China Agricultural University, Guangzhou, People’s Republic of China; b Key Laboratory of Zoonosis Prevention and Control of Guangdong Province, Guangzhou, People’s Republic of China; c Key Laboratory of Veterinary Vaccine Innovation of the Ministry of Agriculture and Rural Affairs, Guangzhou, People’s Republic of China; d National and Regional Joint Engineering Laboratory for Medicament of Zoonosis Prevention and Control, Guangzhou, People’s Republic of China

**Keywords:** H5 subtype, highly pathogenic avian influenza virus, clade 2.3.4.4b, trimeric hemagglutinin, CHO cell

## Abstract

H5 subtype highly pathogenic avian influenza virus (HPAIV) of clade 2.3.4.4b has prevailed globally, causing economic losses in the poultry industry and posing zoonotic threats. While inactivated vaccines are advantageous tools for prevention and control, their reliance on egg-based production and live viruses necessitates safer alternatives. Based on multiple design strategies, including signal peptide replacement, transmembrane domain truncation, fusion with Trimer-tag or *Helicobacter pylori* Ferritin, and adaptation of the EABR [ESCRT (endosomal sorting complex required for transport)- and ALIX (ALG-2-interacting protein X)-binding region] technology, three trimeric hemagglutinin (HA) antigens (S836-Trimer, S836-Ferritin, and S836-ESCRT) were constructed, transiently expressed in HEK293 cells, and the immunogenicity of the candidate antigens was subsequently evaluated through animal studies. Among these, S836-Trimer was identified as optimal based on secretory expression, conformational stability, and immunogenicity. Animal experiments showed that a single immunization with 10 μg of S836-Trimer provided complete protection against a lethal challenge with an epidemic clade 2.3.4.4b virulent strain in chickens, comparable to that of an existing commercial trivalent inactivated vaccine. Subsequently, CHO cell lines stably expressing S836-Trimer were established, achieving ∼10-fold higher antigen yield than HEK293 cells and demonstrating the feasibility of large-scale production of the candidate vaccine. Animal trials confirmed the single-dose efficacy of this CHO cell-derived vaccine. Overall, our research indicates that developing candidate avian influenza vaccines based on mammalian cell expression systems is a promising strategy.

## GRAPHICAL ABSTRACT

**Figure:**
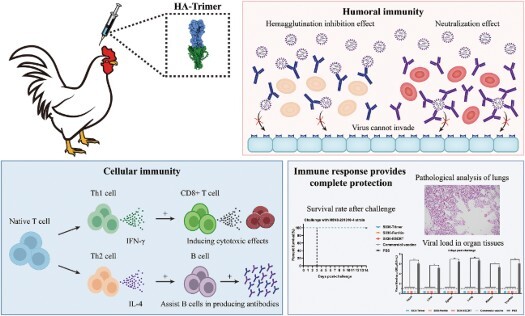

## Introduction

The H5 subtype HPAIV can infect poultry and various wild birds and cause acute death, making it one of the important avian pathogens. Currently, the H5 subtype epidemic strains primarily originate from clade 2.3.4.4, among which 2.3.4.4b is the main epidemic subclade. It is reported that a major wave of the epidemic, primarily caused by H5N8 and H5N1 viruses, has resulted in the death or destruction of hundreds of millions of poultry worldwide, leading to significant economic losses [1]. In addition, the H5 subtype HPAIV also poses a significant threat to human public health [2,3]. Recent studies have shown that a novel H5N1 reassortant virus originating from clade 2.3.4.4b has been detected in dairy cows in the United States [4,5]. Compared to the parental H5N8 virus, this novel reassortant exhibits a stronger ability to overcome species barriers and infect a wider range of mammals, including rodents, cats, minks, seals, and non-human primates [6–9]. Importantly, there have been cases of dairy workers contracting the H5N1 virus through contact with infected cows, the first report of the H5N1 subtype of HPAIV spreading from mammals to humans [10]. These situations collectively indicate the need to take measures to control the spread of HPAIV, especially those originating from clades 2.3.4.4b, in order to prevent their greater impact on poultry farming and human public health safety.

Vaccination is one of the effective measures for preventing and controlling avian influenza. Traditional inactivated vaccines have been widely used, but they still have certain limitations. For example, when a large-scale epidemic occurs, if the supply of chicken embryos is limited, it may cause a delay in the production of chicken embryo-derived vaccines [11]. In addition, whether the production is based on chicken embryos or cells, expensive advanced biosafety laboratories need to be built, so it is urgent to iteratively upgrade traditional vaccines from the perspectives of cost and biosafety. Previous extensive research has shown that genetically engineered subunit vaccines are a new type of vaccine with broad application prospects [12–19]. Currently, research on subunit vaccines for avian influenza mainly focuses on the HA antigen, as it is the main immunogenic protein on the surface of the influenza virus and can induce protective immunity. To ensure the correct folding of exogenous HA proteins into their natural conformation, most studies usually choose to express full-length HA peptide chains. However, the presence of transmembrane domains can cause the HA protein to be embedded on the surface of the cell membrane and unable to be secreted into the cell culture supernatant, which undoubtedly increases the difficulty of harvesting antigens and may increase the production cost of vaccines.

A feasible strategy to resolve the above issues is to replace the transmembrane region of the HA protein with the other domains that can stabilize the trimeric conformation of HA, which could promote the antigen secretion and expression while ensuring the maintenance of the trimeric structure of the HA protein. The H1N1 hemagglutinin vaccine that genetically fused with ferritin derived from *Helicobacter pylori* is a typical example [20]. Trimer-tag, derived from the C-terminal domain of collagen, may be a potential option. Collagen is a triple helix protein composed of three polypeptide chains, and its C-terminal domain plays a crucial role in the correct folding of collagen molecules and the stability of the triple helix structure [21–23]. Research has shown that Trimer-tag derived from the C-terminus of collagen can promote the formation of a homotrimeric structure with its fused tumour necrosis factor-related apoptosis-inducing ligand [24]. Meanwhile, Trimer-tag has also had relevant application examples in the field of vaccine research. Previous research has developed an S-trimer protein vaccine by using Trimer-tag to fuse with the S protein of SARS-CoV-2 that can induce high-level humoral immunity and cellular immune response in the animals, and that candidate COVID-19 vaccine has completed the phase III clinical trial at present, which shows high effectiveness [25]. In addition, the EABR (ESCRT- and ALIX-binding region) technology reported in 2023 has offered a strategy to anchor trimeric S proteins to the surface of enveloped virus-like particles (eVLPs) by recruiting the host ESCRT machinery, thereby promoting budding of S-presenting particles [26]. This approach may point to a new strategy, without requiring protein secretion, anchoring trimeric HA proteins to the surface of enveloped virus-like particles (eVLPs), and the budding process of eVLPs will achieve the goal of carrying trimeric HA proteins to the extracellular environment.

In our study, the HA sequence of the currently epidemic H5 subtype AIV (clade 2.3.4.4b) was used as a template, and based on reasonable modification strategies, which include signal peptide replacement, transmembrane region truncation, fusion of Trimer-tag, or fusion of *Helicobacter pylori* ferritin, and fusion of endocytosis prevention motif (EPM), ESCRT (endosomal sorting complex required for transport)- and ALIX (ALG-2-interacting protein X)-binding region, three different HA antigens were designed. The immunogenicity of these candidate antigens was evaluated through SPF chicken immunization and challenge assays, and the results showed that the candidate HA antigen derived from the HEK293 cells showed potent immunogenicity.

## Materials and methods

### Ethics statement

All experiments involving the highly pathogenic avian influenza virus (HPAIV) were performed in a biosafety level 3 facility at South China Agricultural University in accordance with protocols (CNAS BL0011). All the animal immunization and viral challenge experiments were carried out in accordance with the Guidelines (2017A002) for the Care and Use of Laboratory Animals of South China Agricultural University.

### Bacteria, cells and virus strains

*E. coli* DH5α (Vazyme, Nanjing, China) was used for plasmid DNA production, and the bacteria were cultivated in the LB nutrient medium (Hopebio, Qingdao, China). Human embryo kidney (HEK) 293 cells and CHO-K1 cells were suspension-cultured in shaker flasks with KOP293 medium (Kairui Biotech, Zhuhai, China) and CM-012A medium (Womei Biology, Suzhou, China), respectively, in a 37℃ incubator with 5% CO_2_. Madin-Darby Canine Kidney (MDCK) cells were adherent-cultured in tissue culture dishes with Dulbecco’s modified Eagle’s medium (DMEM) containing 10% (v/v) fetal bovine serum (Gibco, Carlsbad, CA, USA) in a 37℃ incubator with 5% CO_2_. The H5N8 subtype HPAIV A/Duck/Sichuan/201310-4/2020 (H5N8-201310-4, clade 2.3.4.4b) and H5N6 subtype HPAIV A/goose/Guangdong/J565/2025 (H5N6-J565, clade 2.3.4.4b) used in this study were provided by the College of Veterinary Medicine of South China Agricultural University and stored at −80°C. The 50% egg infectious dose (EID_50_) of the virus was calculated using the Reed-Muench method.

### Design and synthesis of genes

**Figure 1.**
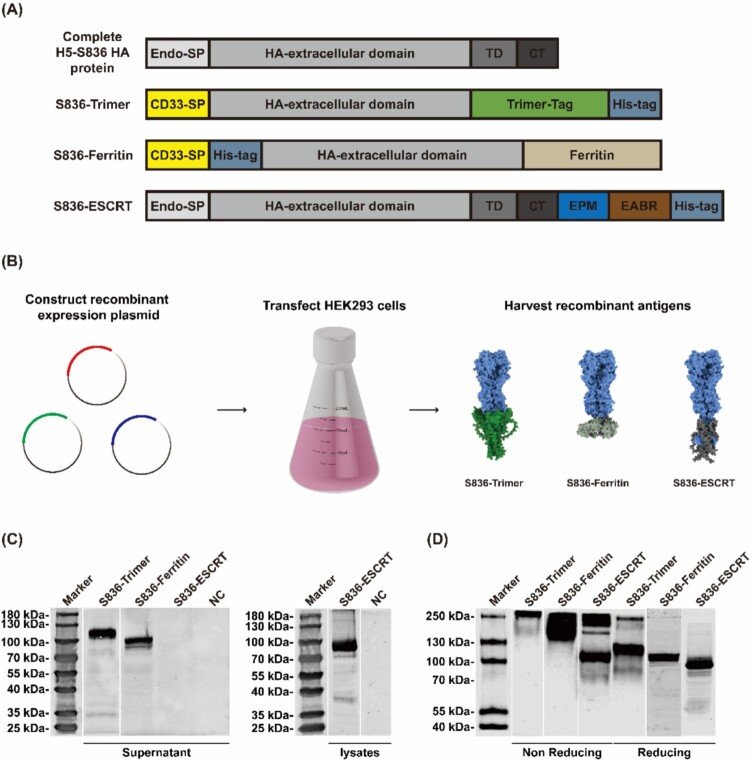
**Preparation and characterization of the candidate HA antigens.** (A) Schematic representations of full-length H5-S836 hemagglutinin (HA), S836-Trimer, S836-Ferritin, and S836-ESCRT protein; SP, signal peptide; TD, transmembrane domain; CT, cytoplasmic tail; Trimer-tag, C-propeptide of collagen; EPM, endocytosis prevention motif; EABR, ESCRT (endosomal sorting complex required for transport)- and ALIX (ALG-2-interacting protein X)- binding region. (B) Schematic diagram of the preparation process of three different candidate H5N8 trimeric HA antigens. (C) Western blot identification of the recombinant HA antigens expression. (D) Trimeric conformation identification of the recombinant HA antigens under non-reducing conditions.

To generate the recombinant trimeric HA antigens, the full-length HA derived from the H5 subtype HPAIV A/Goose/Guangdong/S836/2023 (H5-S836, clade 2.3.4.4b) was used as the template, and three different candidate trimeric HA antigens were constructed based on different trimerization strategies (Figure 1(A)). The S836-Trimer was constructed by fusing the extracellular domain of the HA protein with the Trimer-tag. The S836-Ferritin was constructed by fusing the extracellular domain of the HA protein with the *Helicobacter pylori* ferritin (residues 5-167). And the endogenous signal peptide of the HA protein in the S836-Trimer and the S836-Ferritin was replaced with the CD33 signal peptide to enhance the secretion and expression of proteins. The CD33 signal peptide was selected based on preliminary optimization experiments comparing several signal peptides (including the native HA signal peptide) for secretion efficiency in HEK293 cells (data not shown). The S836-ESCRT was constructed by fusing the full-length HA with the endocytosis prevention motif (EPM), and the ESCRT (endosomal sorting complex required for transport)- and ALIX (ALG-2-interacting protein X)-binding region. For subsequent identification and purification, a 6×His epitope tag was fused with the C-terminus of the S836-Trimer and the S836-ESCRT, as well as the N-terminus of the S836-Ferritin. The amino acid sequences used in this study were summarized in the Supplementary Table 1. All the target genes were codon optimized for the expressing host to achieve higher levels of recombinant protein expression, then biochemically synthesized and respectively cloned into the vector pEE12.4 by Tsingke Biotech Co., Ltd (Beijing, China).

### Preparation and purification of recombinant protein

For the transient expression of recombinant antigens, HEK293 cells were transfected respectively using endotoxin-free recombinant expression plasmids pEE12.4-S836-Trimer, pEE12.4-S836-Ferritin, and pEE12.4-S836-ESCRT via chemical transfection. Briefly, 10 μg plasmid DNA and 50 μg TA-293 reagent (Kairui Biotech, Zhuhai, China) were diluted with KPM transfection buffer (Kairui Biotech, Zhuhai, China), respectively. Then, the diluted TA-293 reagent was mixed with the diluted plasmid DNA and incubated at room temperature for 10 min to produce a plasmid vector complex. Next, the complex solution was gently added into the cells (2.0 × 10^6^ cells/mL), and then the cells were suspension-cultured in a 37℃ incubator with 5% CO_2_ for 72 h to express the recombinant antigens. For the stable expression of recombinant antigens, a linearized endotoxin-free recombinant expression plasmid pEE12.4-S836-Trimer was used to transfect CHO-K1 cells by electroporation, and cell lines stably expressing recombinant proteins were screened by adding L-Methionine sulfoximine (MSX). Next, the cell lines stably expressing the recombinant protein were seeded at a density of 1.0 × 10^6^ cells/mL into cell shake flasks and cultured for 7–10 days to express the recombinant antigen.

The target antigen in the supernatant of the culture medium was purified by nickel affinity chromatography. Briefly, the supernatant was gently incubated with the Ni Focurose FF (VDOBIOTECH, Suzhou, China) equilibrated with phosphate buffer saline (PBS) (pH 7.4) for 4 h at 4℃ to facilitate the binding of the recombinant protein and the Nickel ion. Then, the unbound and non-specific proteins were removed by passing ten column volumes of wash buffer (PBS containing 50 mM Imidazole, pH 7.4). Next, the bound recombinant proteins were eluted from the column using an elution buffer (PBS containing 100 mM Imidazole, pH 7.4), and the eluted fractions containing the protein of interest were dialyzed against PBS (pH 7.4) using a 10 kDa dialysis membrane (Biosharp, Beijing, China). Finally, the expression and purity of the recombinant protein were analyzed using SDS-PAGE and Western blot.

### SDS-PAGE and Western blot

SDS-PAGE and Western blot were used to examine the expression of the recombinant protein as described previously [27]. Briefly, the proteins were denatured by incubating with 5×SDS-PAGE loading buffer (Dingguo, Guangzhou, China) at 100℃ for 10 min. The samples were then separated by electrophoresis on 10% Tris-Glycine gels. For SDS-PAGE analysis, gels were stained with Coomassie Blue Super-Fast Staining Solution (Beyotime, Shanghai, China) for 30 min. For Western blot analysis, proteins were transferred from the gels onto 0.22 µm nitrocellulose membranes, and then blocked with 10% (w/v) skimmed milk for 1 h at room temperature (RT), followed by three washes with PBS. Subsequently, the membranes were incubated overnight at 4℃ with Anti-His-Tag Mouse Monoclonal Antibody (Abbkine, Wuhan, China) diluted at 1:8000. After three washes with PBST [PBS containing 0.05% (v/v) Tween-20], membranes were incubated for 2 h at 4℃ with Goat Anti-Mouse Fluorescence Secondary Antibody (Gene Company Limited, Hong Kong, China) diluted at 1:10000. Finally, membranes were washed three times with PBST and imaged using the Sapphire™ NIR-Q system (Azure Biosystems, Dublin, CA, USA).

### Chicken immunization and challenge studies

Age-matched three-week-old specific pathogen-free (SPF) chickens purchased from Xinxing Dahua Nong Poultry and Egg Co., Ltd (Yunfu, China) were housed in an SPF facility at the Laboratory Animal Center of South China Agricultural University. The programmes of chicken immunization and challenge studies were summarized in the Supplementary Table 2 and Supplementary Table 3.

For the immunogenicity evaluation test of the candidate antigens prepared from HEK293 cells, the SPF chickens were randomly divided into five groups, with 13 chickens in each group. Three groups of chickens were immunized subcutaneously once with 10 μg of S836-Trimer antigens, S836-Ferritin antigens, and S836-ESCRT antigens. All those antigens were formulated with MONTANIDE^TM^ ISA 78 VG. Additionally, as the commercialized vaccines comparison control, another group of chickens was immunized subcutaneously once with the reassortant avian influenza virus (H5 + H7) trivalent inactivated vaccine [(H5N2 rHN5801 Strain + rGD59 Strain, H7N9 rHN7903 Strain), South China Biopharmaceutical, Guangzhou, China], which uses injectable white oil, Span 80, and Tween-80 as adjuvants. As for the unvaccinated control group, the chickens received an equal volume of PBS through the same immunization route. At 21 days post-immunization (dpi), chickens were intranasally challenged with 1.0 × 10^6.0^ EID_50_ (in 200 μL) of the HPAIV H5N8-201310-4, then monitored for clinical symptoms and survival status for 14 serial days. To detect virus shedding, oropharyngeal and cloacal swabs were collected at 3, 5, and 7 days post-challenge (dpc) for virus reisolation in SPF chicken embryos. Additionally, to determine viral replication level in birds, 3 chickens of each group were euthanized at 3 dpc, and the heart, liver, spleen, lung, and trachea were collected for virus titration in SPF chicken embryos. The limit of detection was 1.0 log_10_ EID_50_/0.1 mL for organs. Values below the limit of detection (1.0 log_10_ EID_50_/0.1 mL) were assigned a value of 0.5 log_10_ EID_50_/0.1 mL for statistical analysis.

For the immune efficacy evaluation test of the subunit vaccines based on the antigens from the CHO cells, the SPF chickens were randomly divided into six groups, with 13 chickens in each group. To investigate the immunogenicity of monovalent subunit vaccines at different immunization doses, three groups of chickens were immunized subcutaneously once with 10, 20, and 50 μg of S836-Trimer antigens, respectively. To compare the immunogenicity differences between secretion-type antigens and intracellular-type antigens, another group of chickens was immunized subcutaneously once with 10 μg of S836-Trimer-Cls (cell lysis supernatant, hereinafter referred to as S836-Trimer-Cls). Consistent with before, MONTANIDE^TM^ ISA 78 VG was used as an adjuvant for those antigens. Additionally, one group of chickens was immunized subcutaneously once with the inactivated reassortant avian influenza virus (H5 + H7) trivalent vaccine for the vaccine comparison control. As for the unvaccinated control group, the chickens received an equal volume of PBS through the same immunization route. At 21 days post-immunization (dpi), chickens were intranasally challenged with 1.0 × 10^6.0^ EID_50_ (in 200 μL) of the HPAIV H5N8-201310-4, then monitored for clinical symptoms and survival status for 14 serial days. To detect virus shedding, oropharyngeal and cloacal swabs were collected at 3, 5, and 7 days post-challenge (dpc) for virus reisolation in SPF chicken embryos.

### Vaccine safety assessment

To evaluate the safety of subunit vaccines prepared from antigens expressed by CHO cells in animals, the absorption and inflammatory response of the vaccine at the injection site were observed at 14 and 21 dpi to assess the degree of local side effects of the vaccine. At 19 dpi, three experimental chickens were randomly selected from the S836-Trimer (50 μg) group and the PBS control group, and the trachea, heart, liver, spleen, lungs, kidneys, glandular stomach, and bursa of Fabricius were collected for gross histological observation to further evaluate the safety of the vaccine.

### Serology assays

To determine the efficacy of the vaccines to induce the humoral immune response, the immunized sera were collected, and the hemagglutination inhibition (HI) assay and microneutralization (MN) assay were performed as described previously [27].

For the HI assay, the serially two-fold diluted serum samples were incubated with the 4 hemagglutination units (HAU) of the inactivated H5N8-201310-4 virus antigens at RT for 30 mins in 96-well V-bottom micro reaction plates. Subsequently, 1% (v/v) chicken erythrocyte suspension was added to incubate with the serum-virus mixture at RT for 30 mins. The HI titre was recorded as the reciprocal of the highest serum dilution that fully inhibited the hemagglutination. For the MN assay, serially tenfold diluted serum samples were incubated with 100 median tissue culture infectious doses (TCID_50_) of the H5N8-201310-4 virus at 37°C for 1 h in 96-well cell culture plates. The mixtures were then transferred into monolayer MDCK cells, which were prepared in 96-well cell culture plates, and incubated at 37℃ for 1 h. After incubation, the culture supernatants were replaced by medium supplemented with 2 mg/ml BSA (Dingguo, Guangzhou, China) and 0.5 μg/mL TPCK-trypsin (Dingguo, Guangzhou, China), followed by further incubation for 72 h. Although the H5N8-201310-4 strain is highly pathogenic and possesses a multibasic cleavage site, which allows proteolytic processing independent of exogenous trypsin, TPCK-treated trypsin was supplemented to the culture medium as per standard influenza virus cultivation protocols, as described previously [28–30]. Finally, 50 μL of the cell culture supernatants were diverted into 96-well reaction plates, mixed with an equal volume of 1% (v/v) chicken erythrocyte suspension, and examined for hemagglutination to determine viral presence. The MN antibody titres were defined as the highest dilution that fully neutralized the virus.

### Chicken peripheral blood mononuclear cell (PBMC) and splenocyte isolation and stimulation

At 19 dpi, the peripheral blood and/or spleen tissues from groups of chickens were collected, and the chicken PBMC and/or splenocyte were isolated by using the chicken-specific cell separation kit (Tbdscience, Tianjin, China) according to the manufacturer’s protocols. Then, as described previously [26], the cells isolated were cultured in 6-well cell plates with complete Roswell Park Memorial Institute (RPMI) 1640 medium containing 10% (v/v) FBS and 1% (v/v) Penicillin-Streptomycin Solution (Gibco, Carlsbad, CA, USA). 15 µg of the specific recombinant antigens or inactivated H5N8-201310-4 virus antigens were added to the cells of each well to stimulate, and the cells were incubated for 6 h in a 37℃ incubator with 5% CO_2_. The cells were ultimately collected for RNA extraction and detection of the cytokine expression level.

### RNA extraction and real-time quantitative PCR (RT-qPCR) analysis

To determine the cytokine mRNA expression levels, real-time quantitative PCR (RT-qPCR) was carried out as described previously [26]. Briefly, the total RNA of the stimulated PBMC and/or splenocyte was extracted by using a total RNA rapid extraction kit (Feijie, Shanghai, China). Then, HiScript Reverse Transcriptase (Vazyme, Nanjing, China) was used for the reverse transcription of the RNA sample, 1 μg per sample. Finally, the RT-qPCR was performed using a Bio-Rad CFX96 Deep Well Real-time system PCR instrument (Bio-Rad Laboratories Inc., Hercules, CA, USA) and ChamQ Universal SYBR qPCR master mix (Vazyme, Nanjing, China). The sequences of the primers used in RT-qPCR are shown in Supplementary Table 4. The cytokine levels were normalized to the housekeeping gene β-actin, and the results were reported as the mean value of the fold change compared with the control group.

### Cell proliferation assay

The PBMC isolated were also seeded in 96-well cell plates with complete RPMI-1640 medium for the PBMC proliferation assay. Briefly, the cells were firstly stimulated with or without concanavalin A (con A, Merck, Darmstadt, Germany) at 37℃ for 24 h. Subsequently, the cell proliferation levels were examined by using the MTT cell activity assay kit (RIBOBIO, Guangzhou, China) according to the manufacturer’s instructions. The MTT assay measures cellular metabolic activity, which correlates with the number of viable cells. Therefore, OD_570_ values are used as an indicator of cell proliferation.

### Histological examination

At 3 dpc, lung tissue samples (three of each group) of experimental chickens in each group were collected for histological examination to further evaluate the vaccine's protective efficacy on lung injury caused by viral challenge. All the lung tissue samples were uniformly collected from the middle lobe of the right lung of the chickens, fixed with 4% polyformaldehyde (Dingguo, Guangzhou, China), and then embedded in paraffin. Subsequently, the lung tissue sections were cut to 5 μm thick and stained with hematoxylin and eosin (H&E). By a double-blind method, the histological changes were then examined using a light microscope, and the representative images were captured. The degree of lung injury was scored by multiple pathologists using a double-blind method according to the following criteria: 0 (no obvious pathological changes); 1 (mild lesions: mild structural disorganization of respiratory capillaries or bronchi; mild congestion of vessels, interstitium, or bronchial lumen; mild inflammatory cell infiltration, etc.); 2 (moderate lesions: moderate bronchial or parabronchial dilation with increased lumen area, congestion and/or hemorrhage; moderate interstitial consolidation; marked perivascular or tissue inflammatory cell infiltration, etc.); 3 (severe lesions: severe bronchial dilation, congestion, and/or hemorrhage with red blood cells filling the lumen; epithelial cell necrosis with nuclear fragmentation or irregular arrangement; atrophy and collapse of tertiary bronchi; marked interstitial consolidation, congestion, and/or hemorrhage; extensive inflammatory cell infiltration and focal necrosis in tissues, etc.).

### Statistical analysis

The data analyses were performed and visualized by using GraphPad Prism 9.0 software (GraphPad Software Inc.). Results were expressed as a mean ± standard deviation (SD), and statistical significance was indicated by * (*P* < 0.05), ** (*P* < 0.01), or *** (*P* < 0.001).

## Results

### Construction of candidate HA antigens and the expression in HEK293 cells

Based on different trimerization strategies, three different candidate trimeric HA antigens were constructed (Figure 1(A)). The recombinant expression plasmids were transfected into the HEK293 cells to prepare the recombinant antigens (Figure 1(B)). The expression of the recombinant antigens in HEK293 cells was confirmed by the Western blot (Figure 1(C)). The result showed that the S836-Trimer and S836-Ferritin in a secreted expression form were detected in the culture medium's supernatant. In contrast, no specific protein bands were detected in the supernatant of the culture medium transfected with the S836-ESCRT plasmid, while clear protein bands were detected in the supernatant of lysed cells. The molecular mass of S836-Trimer, S836-Ferritin, and S836-ESCRT was ∼115 kDa, ∼100 kDa, and ∼90 kDa, respectively, which matches expectations. To verify whether the three HA antigens form a trimeric conformation, the protein electrophoresis under non-reducing conditions was conducted (Figure 1(D)). The result showed that all the HA antigens appeared in high-molecular-weight forms corresponding to the trimer. For the S836-ESCRT protein, clear monomeric bands were also detected. The grayscale quantification results showed that the concentration of S836-Trimer antigen was 123.36 μg/mL, the concentration of S836-Ferritin antigen was 95.86 μg/mL (the supernatant was concentrated 10 times), and the concentration of S836-ESCRT antigen was 140.37 μg/mL (Figure S1). The results above showed that the candidate trimeric HA antigens were successfully prepared.

### Candidate HA antigens prepared from HEK293 cells induced potent humoral immunity response

**Figure 2.**
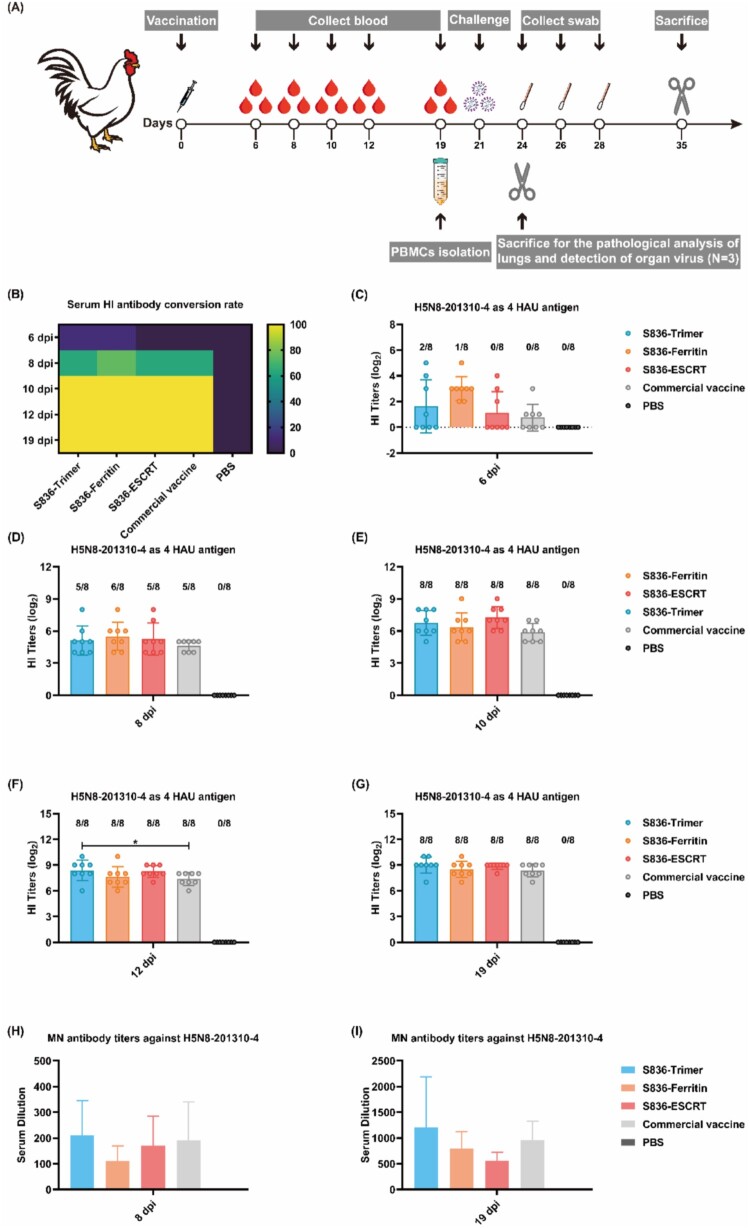
**The immunogenicity evaluation of the candidate HA antigens prepared from HEK293 cells.** (A) Schematic diagram of SPF chicken immunization and challenge experiment process. After the vaccination, the immunized serum was collected to determine the serum antibody levels. The HI antibody titres of the chicken serum were measured with 4 HAU of inactivated H5N8-201310-4 virus antigen at different time points, and the serum positive-conversion rates of each group of animals were calculated (B-G). The neutralizing antibody titres of the chicken serum were measured with 10^2^ TCID_50_ of H5N8-201310-4 virus at 8 dpi (H), and 19 dpi (I). Statistical significance was indicated by * (*P* < 0.05).

To identify the immunogenicity of the candidate trimeric HA antigens, an immunization and challenge study was carried out (Figure 2(A)). SPF chickens were immunized with 10 μg of three different HA antigens prepared from HEK293 cells, formulated with ISA 78 VG, respectively. In addition, existing commercial trivalent inactivated virus vaccines were used as controls. After the vaccination, the HI antibody titre and MN antibody titre were determined to estimate the levels of humoral immune response induced by the candidate antigens.

For the HI antibody titre, there were ∼13% of chickens in the S836-Trimer group and S836-Ferritin group that produced effective HI antibodies (>4 log_2_) at 6 dpi, while the antibody conversion rate of the S836-ESCRT group and the commercial inactivated vaccine groups was still 0% (Figure 2(B and C)). At 8 dpi, most of the chickens in the S836-Trimer group, S836-Ferritin group, S836-ESCRT group, and inactivated vaccine group had produced effective HI antibodies, with antibody conversion rates of 63%, 75%, 63%, and 63%, respectively (Figure 2(B and D)). At 10 dpi, the antibody levels of all vaccine groups had completely converted to positive (Figure 2(B and E)). At 12 dpi, the HI antibody levels of each group were further increased. The average HI antibody titres of the S836-Trimer group and S836-ESCRT group were 8.38 and 8.25 log_2_, respectively (Figure 2(F)), and the average HI antibody titre of the S836-Trimer group was significantly higher than that of the inactivated vaccine group (7.38 log_2_). At 19 dpi, the average antibody titres of all vaccine groups reached over 8 log_2_ (Figure 2(G)). The S836-Trimer group possessed the highest average HI antibody titres of (9 log_2_). For the MN antibody titre, the chickens in each group produced effective MN antibody titres against the H5N8-201310-4 strain (Figure 2(H and I)). The average MN antibody titres of the S836-Trimer group were highest, with 1:210 at 8 dpi and 1:1200 at 19 dpi. The above results indicate that SPF chickens immunized with S836-Trimer can produce higher levels of specific humoral immune response more rapidly.

### Candidate HA antigens prepared from HEK293 cells effectively activated the T-cell immunity response

**Figure 3.**
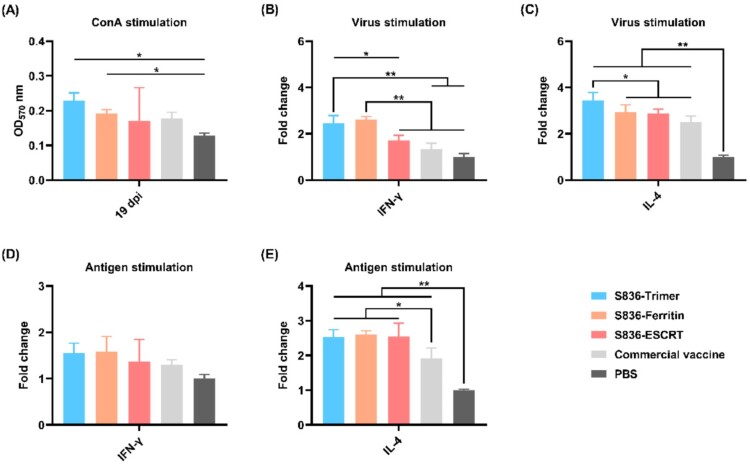
**Proliferation level and cytokine secretion level of chicken PBMC in the immunogenicity evaluation experiment of the candidate HA antigens.** PBMC of chickens from the S836-Trimer group, S836-Ferritin group, S836-ESCRT group, commercial vaccine group, and PBS group were collected at 19 dpi. The proliferation levels of the cells stimulated with Con A were detected using the MTT Cell Viability Assay Kit, and OD_570_ values are used as an indicator of cell proliferation (A). The cells stimulated with specific purified antigens or inactivated virus were used to determine the mRNA expression levels of cytokine IFN-γ (B, D) and IL-4 (C, E) by RT-qPCR. Statistical significance was indicated by * (*P* < 0.05), or ** (*P* < 0.01).

To evaluate the activation of T cell response by recombinant candidate HA antigens, PBMC were isolated from immunized chickens at 19 dpi, stimulated with Con A, and the proliferation level of PBMC in each group was measured by MTT cell activity assay kit. The results showed that, after stimulation with Con A, the proliferation levels of PBMC in the S836-Trimer group and S836-Ferritin group were significantly higher than those in the PBS control group (Figure 3(A)). In addition, compared with the S836-Ferritin group, S836-ESCRT group, and commercial vaccine group, the S836-Trimer group induced the highest level of cell proliferation, but there was no statistical difference between the groups. The above results indicate that immune recombinant candidate antigens can effectively stimulate T cell responses.

To further evaluate the level and type of T cell response induced by recombinant candidate antigens, PBMC collected at 19 dpi were stimulated in vitro, and RNA was extracted. Subsequently, RT-qPCR was used to detect the mRNA expression levels of cytokines IFN-γ and IL-4 associated with Th1 and Th2 immune responses. The results showed that, after the inactivated virus antigen stimulation, effective expression of IFN-γ and IL-4 was detected in PBMC of each vaccine group, with significantly higher average mRNA levels than the control PBS group (Figure 3(B and C)). For the Th1-type response cytokine IFN-γ, the S836-Ferritin group induced the highest mRNA levels, significantly higher than the S836-ESCRT group and the commercial inactivated vaccine group (*P* < 0.01). In addition, the average mRNA level of IFN-γ in the S836-Trimer group was also significantly higher than that in the S836-ESCRT group (*P* < 0.05) and the commercial inactivated vaccine group (*P* < 0.01). For the IL-4, a Th2-type response cytokine, the average mRNA level of the S836-Trimer group was significantly higher than that of the S836-Ferritin group, the S836-ESCRT group, and the commercial inactivated vaccine group (*P* < 0.05). Similar to viral stimulation, effective expression of IFN-γ and IL-4 was also detected in PBMC of the vaccine group after recombinant antigen stimulation (Figure 3(D and E)). For the IFN-γ, the average mRNA levels of each vaccine group were higher than those of the control PBS group, but there was no significant difference between the groups. For the IL-4, the average mRNA levels of each vaccine group were significantly higher than those in the control PBS group (*P* < 0.01). In addition, the average mRNA levels of the S836-Trimer group, S836-Ferritin group, and S836-ESCRT group were significantly higher than those of the commercial inactivated vaccine group (*P* < 0.05).

The above results indicate that the recombinant candidate antigens can effectively activate Th1 and Th2 cell immune responses and induce the secretion of related cytokines. Compared to commercial inactivated vaccines, recombinant candidate antigens can induce higher levels of IFN-γ and IL-4, which may suggest a stronger ability to activate cellular immune responses.

### Candidate HA antigens prepared from HEK293 cells provided chickens with full protection against HPAIV from the epidemic clade 2.3.4.4b

**Figure 4.**
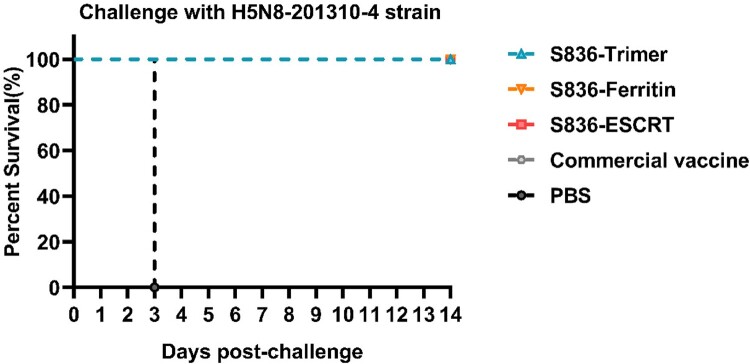
**Survival rates of the chickens after the viral challenge.** At 21 dpi, chickens were intranasally challenged with a lethal dose of HPAIV H5N8-201310-4. After viral challenge, chickens from the vaccine groups survived during the 14-day observation period, while chickens in the PBS group died quickly within three days.

**Figure 5.**
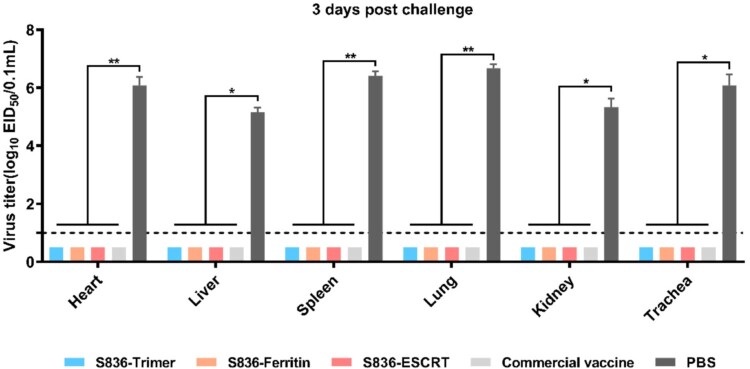
**Virus titres in the organ tissues of the chickens after the viral challenge in the immunogenicity evaluation experiment of the candidate HA antigens.** At 3 dpc, 3 chickens of each group were euthanized to collect the heart, liver, spleen, lung, kidney, and trachea for viral replication level determination. The limit of detection was 1.0 log_10_ EID_50_/0.1 mL for organs. Values below the limit of detection (1.0 log_10_ EID_50_/0.1 mL) were assigned a value of 0.5 log_10_ EID_50_/0.1 mL for statistical analysis. Statistical significance was indicated by * (*P* < 0.05), or ** (*P* < 0.01).

To evaluate the protection efficacy of recombinant candidate HA antigens, all immunized chickens were challenged with a lethal dose of HPAIV H5N8-201310-4 at 21 dpi. The results showed that, the chickens in each vaccine group did not show any symptoms during the entire observation period, and all the survival rates were 100% (Figure 4). As a control, chickens vaccinated with PBS began to develop morbidity and die at 3 days post-challenge (dpc), exhibiting typical flu-like symptoms including difficulty breathing, cyanosis of the crown beard, and central nervous system symptoms. Additionally, oropharynx and cloaca swabs were collected at 3, 5, and 7 dpc to monitor virus shedding. The test results showed that no virus shedding was detected in the chickens of the S836-Trimer group and commercial vaccine group, and the challenge-protective rate against the virus was 100%, while one animal in each of the S836-ESCRT group and the S836-Ferritin group was detected to have virus shedding at 3dpc and 7dpc, respectively, with a challenge-protective rate against the virus of 90% (Table 1). To further investigate the inhibitory effect of vaccination on virus copy in vivo, the organ tissues from chickens were also collected for virus isolation and titration. The detection results showed that the virus was detected in the organ tissues of all PBS-control chickens, with mean titres ranging from 5.2 to 6.7 log_10_ EID_50_/0.1 mL (Figure 5). In contrast, viral titres in the organ tissues of all vaccinated chickens remained below the limit of detection (1.0 log_10_ EID_50_/0.1 mL), and statistical analysis (with values below the detection limit set to 0.5 log_10_ EID_50_/0.1 mL) confirmed that these titres were significantly lower than those in the PBS control group (Figure 5). Generally, the above results indicate that the immune response induced by recombinant candidate antigens could provide the chickens 100% protection against the attack of the HPAIV H5N8-201310-4, avoid clinical symptoms and death, and significantly inhibit virus shedding and replication in vivo.

**Table 1.** Virus shedding of the chickens after the lethal viral challenge in the immunogenicity evaluation experiment of the candidate HA antigens.

Group	Laryngotracheal			Cloacal			Positive swab number/total
	3 dpc	5 dpc	7 dpc	3 dpc	5 dpc	7 dpc	
S836-Trimer	0/10	0/10	0/10	0/10	0/10	0/10	0/10
S836-Ferritin	0/10	0/10	1/10	0/10	0/10	0/10	1/10
S836-ESCRT	1/10	0/10	0/10	1/10	0/10	0/10	1/10
CV	0/10	0/10	0/10	0/10	0/10	0/10	0/10
PBS	NA	NA	NA	NA	NA	NA	NA

CV, commercial vaccine; NA, not applicable due to the death of chickens.

### Candidate HA antigens prepared from HEK293 cells protect chickens from severe pulmonary pathological damage

**Figure 6.**
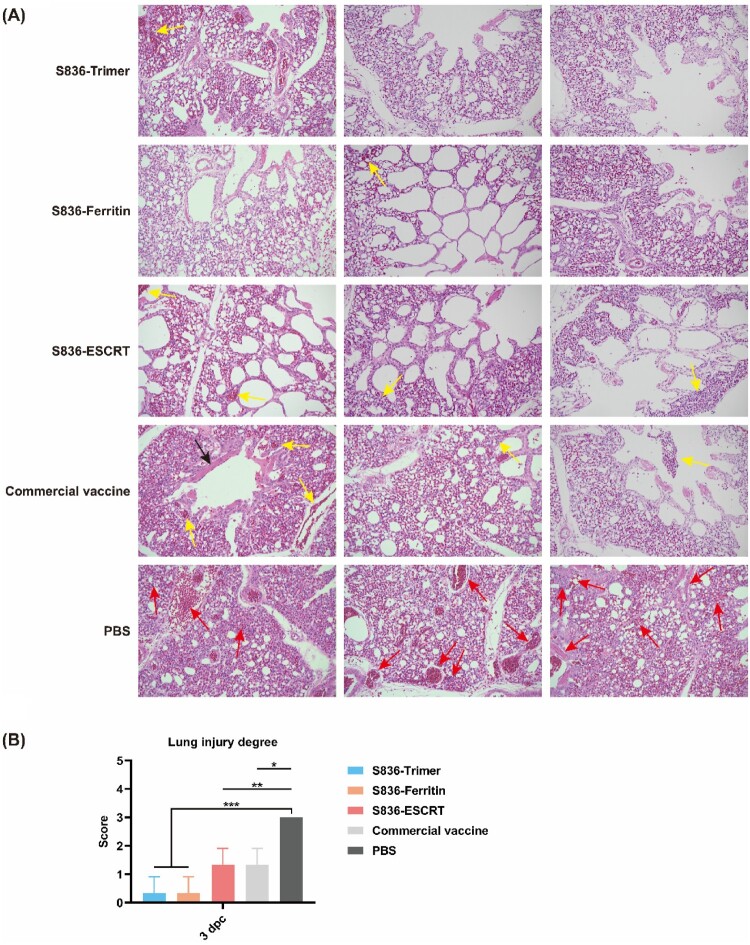
**Evaluation of pathological changes in chicken lung tissue after the viral challenge.** (A) H&E staining of chicken lung tissue sections (three chickens per group, one section per chicken). The yellow arrow indicates mild lesions (including mild structural disorganization of respiratory capillaries or bronchi; mild congestion of vessels, interstitium, or bronchial lumen; mild inflammatory cell infiltration, etc.); the black arrow indicates moderate lesions (including moderate bronchial or parabronchial dilation with increased lumen area, congestion and/or hemorrhage; moderate interstitial consolidation; marked perivascular or tissue inflammatory cell infiltration, etc.); the red arrow indicates severe lesions (including severe bronchial dilation, congestion, and/or hemorrhage with red blood cells filling the lumen; epithelial cell necrosis with nuclear fragmentation or irregular arrangement; atrophy and collapse of tertiary bronchi; marked interstitial consolidation, congestion, and/or hemorrhage; extensive inflammatory cell infiltration and focal necrosis in tissues, etc.). (B) Scoring of chicken lung pathological changes. Statistical significance was indicated by * (*P* < 0.05), ** (*P* < 0.01), or *** (*P* < 0.001).

The influenza virus is a typical respiratory virus that can significantly damage respiratory-related organ tissues. To comprehensively evaluate the protective effect of candidate antigens on the lungs of immunized chickens, the histopathological changes in the lungs of three chickens from each group at 3 dpc were assessed (Figure 6). The results showed that severe pathological changes existed in the lungs of chickens in the PBS control group, including severe bronchial dilation, congestion and hemorrhage, extensive inflammatory cell infiltration, interstitial consolidation, and focal necrosis in tissues (Figure 6(A), red arrows). In contrast, chickens in the vaccine-immunized groups showed only mild or moderate pathological changes. Specifically, only one chicken in each of the S836-Trimer and S836-Ferritin groups exhibited mild lesions, while all three chickens in the S836-ESCRT group showed mild lesions, characterized by mild congestion of blood vessels, interstitium, or bronchial lumen, along with mild inflammatory cell infiltration (Figure 6(A), yellow arrow). Notably, in the commercial vaccine group, one chicken showed moderate lesions, including moderate bronchial dilation and interstitial consolidation (Figure 6(A), black arrow), while the other two chickens showed mild lesions. Quantitative scoring confirmed that the lung injury scores of all vaccine-immunized groups were significantly lower than those of the PBS control group (Figure 6(B)). Meanwhile, the scores of the S836-Trimer, S836-Ferritin, and S836-ESCRT groups were lower than those of the commercial vaccine group, but the differences did not reach statistical significance. Overall, the results demonstrate that the candidate HA antigens effectively protected chickens from severe pulmonary pathological injury induced by viral challenge.

### Expression and identification of the HA-Trimer antigen in stable CHO cells

**Figure 7.**
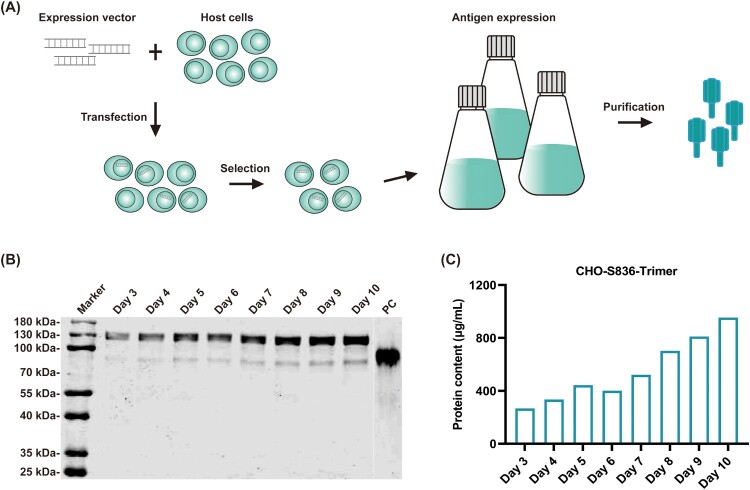
**HA antigen expression and identification in stable CHO cells.** (A) Schematic representations of the antigen expression based on the stable CHO cell expression system. (B) Western blot identification of the feeding batch culture supernatant of stable CHO cells expressing S836-Trimer antigen. (C) Antigen quantification of the feeding batch culture supernatant of stable CHO cells expressing S836-Trimer antigen.

To further explore the feasibility of large-scale production of candidate HA antigens, the stable-transfection CHO cell expression system was used for rapid and large-scale expression of the antigens (Figure 7). Considering the HA-trimer design possesses the highest transient expression level and immunogenicity, the encoding sequence of the S836-Trimer antigen was cloned into a stable expression vector. The recombinant vector carrying the target gene was transfected into CHO-K1 cells by electroporation, and then high-production clones were screened for feeding batch culture, achieving high-level secretion expression of S836-Trimer antigen with a yield of about ∼1 g/L.

### The candidate subunit vaccines based on the HA antigens from the CHO cells possess ideal safety and immune efficacy

At 14 and 21 dpi, the vaccine absorption and inflammatory response at the vaccination site were examined to evaluate the degree of local side effects of the vaccine. The results showed that most of the test chickens absorbed the vaccine well at 14 dpi, and a small amount of unabsorbed white vaccine lotion could be observed at the vaccination site of individual test chickens, but no inflammatory reaction was observed. At 21 dpi, all experimental chickens in each group had fully absorbed the vaccine at the vaccination site, and no inflammatory reactions were observed, indicating that the vaccine had no significant local side effects on the experimental chickens. The absorption of the vaccine was similar to that of the commercial trivalent inactivated vaccine. To further evaluate the safety of the vaccine on experimental chickens, histological examination of major organs was performed on the experimental chickens in the high-dose S836-Trimer (50 μg) group and PBS control group at 19 dpi. The organs of the experimental chickens in the S836-Trimer (50 μg) group showed no abnormal changes, similar to the PBS control group (Figure S2). The above results indicate that the candidate subunit vaccines based on the HA antigens from the CHO cells possess ideal safety.

**Figure 8.**
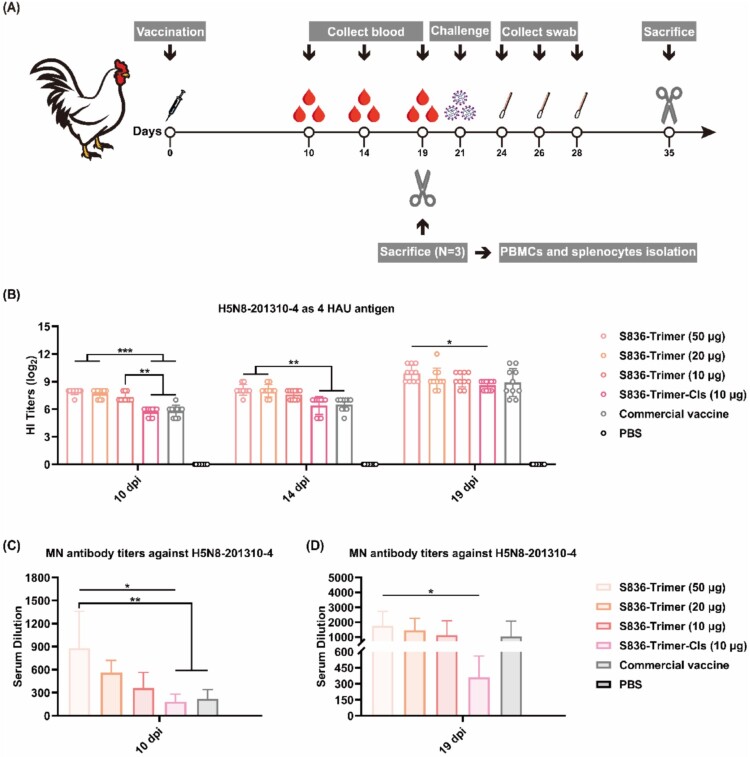
**The immune efficacy evaluation of the candidate subunit vaccine based on CHO cell-expressed antigens.** (A) Schematic diagram of SPF chicken immunization and challenge experiment process. After the vaccination, the immunized serum was collected to determine the serum antibody levels. The HI antibody titres and the MN antibody titres against the H5N8-201310-4 virus of the chicken serum were measured at different time points (B-D). Statistical significance was indicated by ** (*P* < 0.01).

To evaluate the immune efficacy of the candidate subunit vaccine based on CHO cell-expressed antigens, an immunization and challenge study was carried out (Figure 8(A)). After the vaccination, the HI antibody titre and MN antibody titre against the H5N8-201310-4 strain were measured, respectively. Consistent with the antigen expressed in HEK293 cells, the S836-Trimer antigen derived from CHO cells also induced a high HI antibody response. At 10 dpi, except for the S836-Trimer-Cls (10 μg) group (5.7 log_2_) and the commercial vaccine group (5.8 log_2_), the average HI antibody titres of the other groups were all over 7 log_2_, which were significantly higher than the former (Figure 8(B)). At 19 dpi, the average antibody levels of each group further increased, all reaching over 8 log_2_, and the average antibody levels of the S836-Trimer (50 μg) group, S836-Trimer (20 μg) group, and S836-Trimer (10 μg) group were all over 9 log_2_. As for the MN antibody level, at 10 dpi, the average antibody titre of the S836-Trimer (10 μg) group reached 1:360, which is 1.63 times that of the commercial vaccine group and twice that of the S836-Trimer-Cls (10 μg) group at the same dose (Figure 8(C)). At 19 dpi, the average MN antibody titre of the S836-Trimer (10 μg) group reached 1:1120, which was 3.11 times higher than that of the S836-Trimer-Cls group at the same dose (Figure 8(D)). The results indicate that the secreted S836-Trimer antigen derived from CHO cells possesses stronger immunogenicity, and immunization with 10 μg of antigen can induce significantly stronger humoral immunity response in a shorter time (10 dpi) than equivalent doses of S836-Trimer cell lysis antigen and commercial vaccines. Meanwhile, we have also performed cross-neutralization assays against the novel variant (H5N6-J565 strain) isolated in 2025 using the collected sera, and the results showed that effective neutralizing antibodies were still detectable (Figure S3), indicating that the vaccine developed in this study has potential cross-protective efficacy against the novel variant.

Additionally, cytokine secretion levels in PBMC and splenocyte from the S836-Trimer (10 μg) group, the commercial vaccine group, and the PBS group were measured at 19 dpi to assess cellular immune responses. As shown in Figure S4, the mRNA levels of IFN-γ and IL-4 in the S836-Trimer group were significantly higher than those in the PBS group, which indicate that the subunit vaccines could effectively activate Th1 and Th2 cell immune responses.

### Immune protection efficacy of the candidate subunit vaccines based on the antigens from the CHO cells

**Figure 9.**
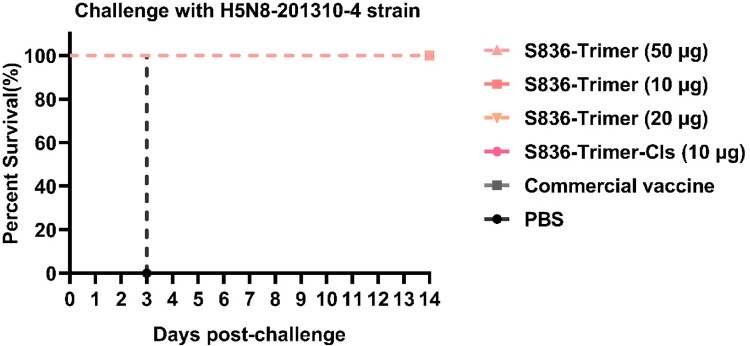
**Survival rates of the chickens after the viral challenge in the immune efficacy evaluation experiment of the candidate subunit vaccine based on CHO cell-expressed antigens**. At 21 dpi, chickens were intranasally challenged with a lethal dose of HPAIV H5N8–201310-4. After the viral challenge, chickens from the vaccine groups survived during the 14-day observation period, while chickens in the PBS group died quickly within three days.

To evaluate the protection efficacy of the candidate subunit vaccines based on the antigens from the CHO cells, the immunized chickens were challenged with a lethal dose of HPAIV H5N8-201310-4. The results showed that the chickens in each vaccine group survived during the whole observation period without any symptoms (Figure 9). As a control, the chickens receiving PBS began to develop symptoms and die at 3 dpc. The results of the virus shedding test showed that, except for one chicken in the S836-Trimer-Cls (10 μg) group, which was detected to have virus shedding at 3dpc, no virus shedding was detected in the chickens of the other vaccine groups (Table 2). The above results indicate that the subunit vaccine possesses sufficient immune efficacy, and 10 μg of S836-Trimer antigen can provide 100% clinical protection against lethal attacks of the epidemic virulent strain H5N8-201310-4 in chickens, and completely inhibits virus shedding, providing 100% challenge protection.

**Table 2.** Virus shedding of the chickens after the challenge of the H5N8-201310-4 strain in the immune efficacy evaluation experiment of the candidate subunit vaccine based on CHO cell-expressed antigens.

Group	Laryngotracheal		Cloacal		Positive swab number/total
	3 dpc	5 dpc	3 dpc	5 dpc	
S836-Trimer (50 μg)	0/10	0/10	0/10	0/10	0/10
S836-Trimer (20 μg)	0/10	0/10	0/10	0/10	0/10
S836-Trimer (10 μg)	0/10	0/10	0/10	0/10	0/10
S836-Trimer-Cls (10 μg)	1/10	0/10	0/10	0/10	1/10
CV	0/10	0/10	0/10	0/10	0/10
PBS	NA	NA	NA	NA	NA

Cls, cell lysis supernatant; CV, commercial vaccine; NA, not applicable due to the death of chickens.

## Discussions

The frequent mutations of H5 subtype AIVs pose a persistent threat, especially the 2024 incident of cow-derived H5N1 subtype AIVs infecting humans in the United States, which further exacerbates widespread concerns worldwide [31]. For a long time, the immune prevention and control of avian influenza in China had highly relied on the use of inactivated virus vaccines [32]. Undoubtedly, this vaccine has played an indelible and important role in the prevention and control of avian influenza in China, but its limitations highlight the necessity of developing new vaccines.

The HA subunit vaccine of avian influenza virus has been widely studied and is considered a promising candidate for the next generation of avian influenza vaccines [33–35]. The HA protein is the key to the successful invasion of host cells by the influenza virus. Influenza virus recognizes receptors on host cells through the sialic acid receptor binding site at the head of the HA protein, and initiates the virus infection cycle through membrane fusion mediated by a fusion peptide located at the N-terminus of the HA2 subunit [36]. In the protective immunity against the influenza virus, neutralizing antibodies mainly target key epitopes on the HA protein head (such as receptor binding sites), blocking the binding of virus HA to host cell surface sialic acid, thereby inhibiting virus invasion [37]. Therefore, the HA protein is the most important target in the development and research of influenza vaccines. The natural HA protein is a trimeric protein formed by three homologous monomeric HA molecules, and its immunogenicity is highly correlated with its trimeric conformation [38]. In addition, studies have shown that certain neutralizing epitopes of the HA protein rely on its trimeric spatial conformation [39,40]. The trimeric conformation of the HA protein is maintained jointly by the interaction between the transmembrane α-helix coiled-coil, stem α-helix coiled-coil, and head-specific residues. Among them, the α-helix structure in the transmembrane region is considered to be the initiating factor for the formation of trimeric conformation in the HA protein. Therefore, to maintain the complete trimeric conformation of the HA protein, most studies choose to express full-length HA peptide chains when expressing HA antigens. However, this will cause the expressed HA protein to be localized on the cell membrane surface and unable to be secreted into the culture supernatant, which may complicate the vaccine development process and increase production costs.

In our study, different combination strategies were used to design the HA protein to form a trimeric conformation and simultaneously secrete it into the culture supernatant (Figure 1(A)). Firstly, the endogenous signal peptide of the HA protein was replaced with the more efficient exogenous CD33 signal peptide, which was used to enhance the secretion and expression of proteins in CHO cells. Next, the three-dimensional structure of the HA protein was visualized using AlphaFold3, and the truncation site was selected. The core hydrophobic part of the transmembrane domain of the HA protein and its subsequent cytoplasmic tail region were truncated at the position where maintaining the extracellular domain forms a complete conformation, based on structural alignment with the full-length HA model, achieving minimal structural disruption (RMSD below 1.0 Å). Finally, Trimer-tag and *Helicobacter pylori* ferritin were respectively genetically fused to the C-terminus of the truncated HA protein, both of which have been reported to promote the correct formation of the trimeric conformation of the HA protein. In addition, we also designed the HA-ESCRT antigen based on the EABR technology reported in 2023 [26]. This technology involves incorporating EABR (ESCRT- and ALIX binding region) motifs into the C-terminus of the full-length membrane protein of the virus, using the internal sorting complex (ESCRT) pathway required for transport to recruit host proteins and assemble them into enveloped virus-like particles (eVLPs) for budding. The HA protein on the surface of these particles will form the correct trimeric conformation, similar to the HA protein on the surface of natural virus particles. The subsequent protein expression results showed that S836-Trimer antigen and S836-Ferritin antigen existed in the cell culture supernatant in a secreted and trimeric form (Figure 1(C and D)), but the secretion yield of S836-Ferritin antigen was significantly lower than that of S836-Trimer antigen (Figure S1). We acknowledge that non-reducing SDS-PAGE, while suggestive of trimer formation, does not definitively confirm the native trimeric conformation. Future studies incorporating size-exclusion chromatography or blue native PAGE would provide more rigorous biophysical characterization of the trimeric state. Importantly, the robust neutralizing antibody responses elicited by the S836-Trimer antigen provide functional evidence that the antigen was presented in its native trimeric conformation, as neutralizing epitopes on influenza HA are highly conformation-dependent. For the S836-ESCRT antigen, unlike previous studies, the target protein band in the culture supernatant of transfected cells was not detected (Figure 1(C)), indicating the absence of budding eVLPs. The result may be attributed to the low efficiency of the EABR motif that fuses to the C-terminus of the HA protein in recruiting host proteins. In addition, it may also be related to the characteristics of different membrane proteins, especially their transmembrane regions, which can be supported by another group's research [41,42]. Their studies found that SARS-CoV-2 eVLPs can be produced from mammalian cells by overexpression of full-length S protein alone, without any other viral components or exogenous motifs. However, no eVLPs were detected when overexpressing the full-length HA protein. Given that the S836-ESCRT antigen could also form a trimeric conformation (Figure 1(D)), we conducted subsequent immunogenicity evaluation experiments with the lysate of transfected cells and other secretion-type candidate antigens together.

The immunogenicity evaluation results showed that all three candidate HA antigens exhibited potent immunogenicity, with SPF chickens producing high levels of HI and MN antibody responses upon receiving a single dose of 10 μg of immunization (Figure 2). Among them, the S836-Trimer antigen showed superiority. Notably, chickens immunized with the S836-Trimer and S836-Ferritin antigens mounted detectable HI antibody responses as early as 6 dpi, earlier than typically observed with inactivated vaccines, suggesting that this characteristic may represent an advantage of the subunit platform. This rapid seroconversion may be attributed to the native-like trimeric conformation of the antigen, which preserves critical neutralizing epitopes, and to the sustained-release properties of the ISA 78 VG adjuvant [43]. Importantly, the S836-Trimer antigen provided significant immune protection to immunized chickens, fully resisting lethal attacks from the virulent strain from the clade 2.3.4.4b (Table 1). No viral titres were detected in various tissues and organs (Figure 5), and no severe pathological damage was observed in the lungs of the experimental chickens (Figure 6), indicating that virus replication was significantly inhibited. In summary, the immune efficacy of the candidate antigen is equivalent to the existing commercially available inactivated vaccines. These results indicate that it is feasible to prepare candidate trimeric HA antigens using HEK293 cells.

The production cost of vaccines is a key factor to consider when developing new-generation candidate vaccines. To further enhance the expression level of candidate antigens and explore the feasibility of large-scale production of this mammalian cell-derived candidate avian influenza subunit vaccine, the recombinant CHO cells that stably express the candidate antigens were constructed based on the stable-transfection CHO cell expression system (Figure 7). CHO cells are considered to possess high recombinant gene amplification and protein secretion expression abilities, and are suitable for large-scale high-density suspension culture to prepare recombinant protein [44,45]. In our study, compared to using HEK293 cells for recombinant antigen expression, the expression yield of recombinant CHO cells stably expressing antigens was increased by nearly ten times, and it is expected to be further improved by optimizing gene vectors and cell culture techniques, etc [46].

Subsequently, the subunit vaccines based on stable cell expression antigens were prepared, and the immune efficacy of the vaccines was comprehensively evaluated through SPF chicken immunization and challenge trials. Consistent with previous vaccines based on HEK293 cells, vaccines derived from CHO cells also exhibited ideal immune efficacy. Specifically, the immunogenicity of vaccines at different antigen doses (50, 20, 10 μg) was explored (Figure 8). The results showed that antibody levels were dose-dependent, with the 50 μg group inducing the highest levels of HI and MN antibodies. However, there was no statistically significant difference in antibody levels between the 50 μg group and the 10 μg group at 19 dpi (Figure 8(B and C)). Importantly, the two groups provided equivalent protection against viral attacks, indicating that 10 μg is the ideal antigen dose. Meanwhile, the immunogenicity differences between secretion-type S836-Trimer antigen and intracellular-type S836-Trimer antigen were also compared, and the results showed that secretion-type S836-Trimer antigen possesses superior immunogenicity, inducing higher antibody levels, especially at the early time point after immunization (10 dpi), when HI antibody levels were significantly higher than intracellular-type S836-Trimer antigen (Figure 8(B)). Interestingly, despite originating from the same cells, the molecular weight of the intracellular-type S836-Trimer antigen is significantly lower than that of the secretion-type S836-Trimer antigen (data not shown), which may be related to its lower degree of glycosylation, and these results once again suggests that glycosylation may affect the immunogenicity of the antigen [47–50].

As expected, the candidate subunit vaccine provided 100% immune protection to immunized chickens, completely resisting the lethal challenge of the epidemic virulent strain (Figure 9, Table 2). The results above indicate that the candidate subunit vaccines prepared from CHO cell-expressed antigens possess strong immune efficacy. Several previous studies have reported the development of recombinant HA-based subunit vaccines against avian influenza using CHO cell expression systems. For instance, Chen et al. developed an H7N9 subunit vaccine using CHO cells, but required two immunizations of 15 μg antigen to achieve full protection [33]. Similarly, Zhu et al. reported an H9 subunit vaccine based on CHO cells that also required a prime-boost regimen [51]. In contrast, our S836-Trimer vaccine achieved complete protection with a single 10 μg dose, representing a significant advantage in terms of dose-sparing and logistical simplicity. Additionally, compared to baculovirus-insect cell expression systems (e.g. Flublok®), which have been used for seasonal influenza vaccines, our CHO cell platform offers several potential advantages: (i) higher expression yield (∼1 g/L) compatible with large-scale industrial production; (ii) absence of baculovirus-related genetic instability; (iii) well-established regulatory framework for CHO cell-derived biopharmaceuticals. Importantly, the complete protection achieved in the S836-Trimer group, characterized by complete inhibition of virus shedding and replication, distinguishes this vaccine from many previous subunit vaccine candidates that only provided clinical protection without preventing transmission.

In China, the implementation of a nationwide vaccination programme using bivalent (H5/H7) inactivated vaccines has successfully controlled the spread of HPAIV since 2017 [32]. However, the reliance on embryonated chicken eggs for vaccine production poses challenges during large-scale outbreaks when egg supply may be limited. Moreover, the production process requires high-containment facilities. Compared with the inactivated vaccines currently used in China, our mammalian cell-derived subunit vaccine offers a potential alternative that bypasses these limitations while providing comparable or even slightly stronger protective efficacy to the commercial inactivated vaccine.

Meanwhile, we acknowledge several limitations in this study. First, cytokine responses were assessed only at the mRNA level, and future studies incorporating protein-level validation (e.g. ELISA) would provide a more complete picture of the cellular immune response. Second, we measured only IFN-γ and IL-4 as representatives of Th1 and Th2 responses; a broader panel of cytokines, including Th17-related cytokines (e.g. IL-17) and T cell activation markers, would provide a deeper understanding of the cellular immunity induced. Third, the vaccine candidate was developed based on a clade 2.3.4.4b strain isolated in 2023, and we demonstrated its protective efficacy against a homologous challenge. However, antigenic drift and the emergence of new subclades are ongoing concerns. Although the cross-neutralization assay indicated that the vaccine immune sera possessed effective cross-neutralizing activity (Figure S3), the cross-protective efficacy of the vaccine against variant strains still needs to be rigorously verified by future challenge studies.

In conclusion, we designed and prepared three candidate trimeric H5 subtype HA antigens and found that those derived from HEK293 cells exhibited ideal immunogenicity in animal experiments, inducing both humoral and cellular immune responses to protect chickens against lethal challenges from the epidemic virulent strain. Subsequently, the HA-Trimer antigen with the highest transient expression level and immunogenicity was selected to construct stable-transfection CHO cells using the stable-transfection CHO cell system, and the subunit vaccines were prepared based on these antigens. The vaccine also demonstrated strong immune efficacy in animals, and the efficacy of 10 μg of effective antigen was comparable to that of the existing commercially available inactivated vaccines. Unlike traditional inactivated vaccines, the development process of this candidate vaccine does not require the cultivation of live viruses and possesses higher safety. Our research results indicate that developing novel candidate avian influenza vaccines based on the mammalian cell expression system, especially the CHO cell expression system, is a promising strategy.

## Supplementary Material

Supplementary material.docx

## References

[1] Shi J, Zeng X, Cui P, et al. Alarming situation of emerging H5 and H7 avian influenza and effective control strategies. Emerg Microbes Infect. 2023;12(1):2155072. doi:10.1080/22221751.2022.2155072 36458831 PMC9754034

[2] Gu W, Shi J, Cui P, et al. Novel H5N6 reassortants bearing the clade 2.3.4.4b HA gene of H5N8 virus have been detected in poultry and caused multiple human infections in China. Emerg Microbes Infect. 2022;11(1):1174–1185. doi:10.1080/22221751.2022.2063076 35380505 PMC9126593

[3] Zhang Y, Wu J, Lin Q, et al. Infection tracing and virus genomic analysis of two cases of human infection with avian influenza A(H5N6) – Fujian province, China, April–May 2024. China CDC Wkly. 2025;7(3):107–112. doi:10.46234/ccdcw2024.274 39867819 PMC11757903

[4] Caserta LC, Frye EA, Butt SL, et al. Spillover of highly pathogenic avian influenza H5N1 virus to dairy cattle. Nature. 2024;634(8034):669–676. doi:10.1038/s41586-024-07849-4 39053575 PMC11485258

[5] Mostafa A, Naguib MM, Nogales A, et al. Avian influenza A (H5N1) virus in dairy cattle: origin, evolution, and cross-species transmission. mBio. 2024;15(12):e254224. doi:10.1128/mbio.02542-24 PMC11633217 39535188

[6] Burrough ER, Magstadt DR, Petersen B, et al. Highly pathogenic avian influenza A(H5N1) clade 2.3.4.4b virus infection in domestic dairy cattle and cats, United States, 2024. Emerg Infect Dis. 2024;30(7):1335–1343. doi:10.3201/eid3007.240508 38683888 PMC11210653

[7] Restori KH, Septer KM, Field CJ, et al. Risk assessment of a highly pathogenic H5N1 influenza virus from mink. Nat Commun. 2024;15(1):4112. doi:10.1038/s41467-024-48475-y 38750016 PMC11096306

[8] Tipih T, Mariappan V, Yinda KC, et al. Highly pathogenic avian influenza H5N1 clade 2.3.4.4b genotype B3.13 is highly virulent for mice, rapidly causing acute pulmonary and neurologic disease. Nat Commun. 2025;16(1):5738. doi:10.1038/s41467-025-60407-y 40593471 PMC12216816

[9] Rosenke K, Griffin A, Kaiser F, et al. Pathogenesis of bovine H5N1 clade 2.3.4.4b infection in macaques. Nature. 2025;640(8060):1017–1021. doi:10.1038/s41586-025-08609-8 39814072

[10] Garg S, Reed C, Davis CT, et al. Outbreak of highly pathogenic avian influenza A(H5N1) viruses in U.S. dairy cattle and detection of two human cases – United States, 2024. MMWR Morb Mortal Wkly Rep. 2024;73(21):501–505. doi:10.15585/mmwr.mm7321e1 38814843 PMC11152367

[11] Quan FS, Lee YT, Kim KH, et al. Progress in developing virus-like particle influenza vaccines. Expert Rev Vaccines. 2016;15(10):1281–1293. doi:10.1080/14760584.2016.1175942 27058302 PMC5093318

[12] Liu G, Zhang F, Shi J, et al. A subunit vaccine candidate derived from a classic H5N1 avian influenza virus in China protects fowls and BALB/c mice from lethal challenge. Vaccine. 2013;31(46):5398–5404. doi:10.1016/j.vaccine.2013.09.009 24055355

[13] Dunkle LM, Izikson R, Patriarca PA, et al. Randomized comparison of immunogenicity and safety of quadrivalent recombinant versus inactivated influenza vaccine in healthy adults 18–49 years of age. J Infect Dis. 2017;216(10):1219–1226. doi:10.1093/infdis/jix478 28968871

[14] Richards KA, Moritzky S, Shannon I, et al. Recombinant HA-based vaccine outperforms split and subunit vaccines in elicitation of influenza-specific CD4 T cells and CD4 T cell-dependent antibody responses in humans. Npj Vaccines. 2020;5:77. doi:10.1038/s41541-020-00227-x 32884842 PMC7450042

[15] Honda-Okubo Y, Bart TE, Hurst BL, et al. An Advax-CpG adjuvanted recombinant H5 hemagglutinin vaccine protects mice against lethal influenza infection. Vaccine. 2023;41(39):5730–5741. doi:10.1016/j.vaccine.2023.08.009 37567799

[16] Zhang X, Zhang F, Chen N, et al. A rationally designed H5 hemagglutinin subunit vaccine provides broad-spectrum protection against various H5Nx highly pathogenic avian influenza viruses in chickens. Vaccines (Basel). 2024;12(8):932. doi:10.3390/vaccines12080932 39204055 PMC11359994

[17] Feng J, Du Y, Chen L, et al. A quadrivalent recombinant influenza hemagglutinin vaccine induced strong protective immune responses in animal models. Vaccine. 2024;42(22):126008. doi:10.1016/j.vaccine.2024.05.056 38834431

[18] He Y, Wang J, Chi L, et al. Combination adjuvants enhance recombinant H5 hemagglutinin vaccine protection against high-dose viral challenge in chickens. Vaccines (Basel). 2024;12(12):1448. doi:10.3390/vaccines12121448 39772109 PMC11680309

[19] Hegmann TE, Walter EB, Smith MJ, et al. A phase I study of the safety, reactogenicity and immunogenicity of two quadrivalent seasonal influenza vaccines (Fluzone(R) or Flublok(R)) with or without one of two adjuvants (AF03 or Advax-CpG55.2) in healthy adults 18-45 years of age. Vaccine. 2025;54:126991. doi:10.1016/j.vaccine.2025.126991 40107003

[20] Kanekiyo M, Wei CJ, Yassine HM, et al. Self-assembling influenza nanoparticle vaccines elicit broadly neutralizing H1N1 antibodies. Nature. 2013;499(7456):102–106. doi:10.1038/nature12202 23698367 PMC8312026

[21] Bernocco S, Finet S, Ebel C, et al. Biophysical characterization of the C-propeptide trimer from human procollagen III reveals a tri-lobed structure. J Biol Chem. 2001;276(52):48930–48936. doi:10.1074/jbc.M108611200 11684689

[22] Khoshnoodi J, Cartailler JP, Alvares K, et al. Molecular recognition in the assembly of collagens: terminal noncollagenous domains are key recognition modules in the formation of triple helical protomers. J Biol Chem. 2006;281(50):38117–38121. doi:10.1074/jbc.R600025200 17082192

[23] Boudko SP, Engel J, Bachinger HP. The crucial role of trimerization domains in collagen folding. Int J Biochem Cell Biol. 2012;44(1):21–32. doi:10.1016/j.biocel.2011.09.009 22001560

[24] Liu H, Su D, Zhang J, et al. Improvement of pharmacokinetic profile of TRAIL via trimer-tag enhances its antitumor activity in vivo. Sci Rep. 2017;7(1):8953. doi:10.1038/s41598-017-09518-1 28827692 PMC5566391

[25] Liang JG, Su D, Song TZ, et al. S-Trimer, a COVID-19 subunit vaccine candidate, induces protective immunity in nonhuman primates. Nat Commun. 2021;12(1):1346. doi:10.1038/s41467-021-21634-1 33649323 PMC7921634

[26] Hoffmann M, Yang Z, Huey-Tubman KE, et al. ESCRT recruitment to SARS-CoV-2 spike induces virus-like particles that improve mRNA vaccines. Cell. 2023;186(11):2380–2391. doi:10.1016/j.cell.2023.04.024 37146611 PMC10121106

[27] Chen T, Gao Y, Chen X, et al. Self-assembling nanoparticle vaccine elicits a robust protective immune response against avian influenza H5N6 virus in chickens. Int J Biol Macromol. 2025;287:138405. doi:10.1016/j.ijbiomac.2024.138405 39643188

[28] WWHO Global Influenza Surveillance Network. Manual for the laboratory diagnosis and virological surveillance of influenza. Geneva: World Health Organization; 2011.

[29] Choi WS, Baek YH, Kwon JJ, et al. Rapid acquisition of polymorphic virulence markers during adaptation of highly pathogenic avian influenza H5N8 virus in the mouse. Sci Rep. 2017;7:40667. doi:10.1038/srep40667 28094780 PMC5240553

[30] Ahrens AK, Selinka HC, Mettenleiter TC, et al. Exploring surface water as a transmission medium of avian influenza viruses – systematic infection studies in mallards. Emerg Microbes Infect. 2022;11(1):1250–1261. doi:10.1080/22221751.2022.2065937 35473641 PMC9090351

[31] Abbasi J. Bird Flu outbreak in dairy cows is widespread, raising public health concerns. JAMA. 2024;331(21):1789–1791. doi:10.1001/jama.2024.8886 38718040

[32] Zeng X, Shi J, Chen H. Control of highly pathogenic avian influenza through vaccination. J Integr Agr. 2024;23(5):1447–1453. doi:10.1016/j.jia.2024.03.044

[33] Chen TH, Liu WC, Chen IC, et al. Recombinant hemagglutinin produced from Chinese Hamster Ovary (CHO) stable cell clones and a PELC/CpG combination adjuvant for H7N9 subunit vaccine development. Vaccine. 2019;37(47):6933–6941. doi:10.1016/j.vaccine.2019.02.040 31383491 PMC7115541

[34] Lin TH, Chia MY, Lin CY, et al. Improving immunogenicity of influenza virus H7N9 recombinant hemagglutinin for vaccine development. Vaccine. 2019;37(13):1897–1903. doi:10.1016/j.vaccine.2018.09.034 30857635

[35] Lu Y, Landreth S, Liu G, et al. Innate immunemodulator containing adjuvant formulated HA based vaccine protects mice from lethal infection of highly pathogenic avian influenza H5N1 virus. Vaccine. 2020;38(10):2387–2395. doi:10.1016/j.vaccine.2020.01.051 32014270

[36] Brown EG. Influenza virus genetics. Biomed Pharmacother. 2000;54(4):196–209. doi:10.1016/S0753-3322(00)89026-5 10872718

[37] Padilla-Quirarte HO, Lopez-Guerrero DV, Gutierrez-Xicotencatl L, et al. Protective antibodies against influenza proteins. Front Immunol. 2019;10:1677. doi:10.3389/fimmu.2019.01677 31379866 PMC6657620

[38] Weldon WC, Wang BZ, Martin MP, et al. Enhanced immunogenicity of stabilized trimeric soluble influenza hemagglutinin. PLoS One. 2010;5(9):e12466. doi:10.1371/journal.pone.0012466 20824188 PMC2931692

[39] Krammer F, Margine I, Tan GS, et al. A carboxy-terminal trimerization domain stabilizes conformational epitopes on the stalk domain of soluble recombinant hemagglutinin substrates. PLoS One. 2012;7(8):e43603. doi:10.1371/journal.pone.0043603 22928001 PMC3426533

[40] Magadan JG, Khurana S, Das SR, et al. Influenza A virus hemagglutinin trimerization completes monomer folding and antigenicity. J Virol. 2013;87(17):9742–9753. doi:10.1128/JVI.00471-13 23824811 PMC3754138

[41] Alpuche-Lazcano SP, Stuible M, Akache B, et al. Preclinical evaluation of manufacturable SARS-CoV-2 spike virus-like particles produced in Chinese Hamster Ovary cells. Commun Med (Lond). 2023;3(1):116. doi:10.1038/s43856-023-00340-7 37612423 PMC10447459

[42] Sanchez-Martinez ZV, SP A-L, Stuible M, et al. SARS-CoV-2 spike-based virus-like particles incorporate influenza H1/N1 antigens and induce dual immunity in mice. Vaccine. 2024;42(26):126463. doi:10.1016/j.vaccine.2024.126463 39481241

[43] Aucouturier J, Dupuis L, Ganne V. Adjuvants designed for veterinary and human vaccines. Vaccine. 2001;19(17-19):2666–2672. doi:10.1016/S0264-410X(00)00498-9 11257407

[44] Kim JY, Kim YG, Lee GM. Cho cells in biotechnology for production of recombinant proteins: current state and further potential. Appl Microbiol Biotechnol. 2012;93(3):917–930. doi:10.1007/s00253-011-3758-5 22159888

[45] Lai T, Yang Y, Ng SK. Advances in Mammalian cell line development technologies for recombinant protein production. Pharmaceuticals (Basel). 2013;6(5):579–603. doi:10.3390/ph6050579 24276168 PMC3817724

[46] You M, Yang Y, Zhong C, et al. Efficient mAb production in CHO cells with optimized signal peptide, codon, and UTR. Appl Microbiol Biotechnol. 2018;102(14):5953–5964. doi:10.1007/s00253-018-8986-5 29740673

[47] Wei CJ, Xu L, Kong WP, et al. Comparative efficacy of neutralizing antibodies elicited by recombinant hemagglutinin proteins from avian H5N1 influenza virus. J Virol. 2008;82(13):6200–6208. doi:10.1128/JVI.00187-08 18417563 PMC2447076

[48] Wang CC, Chen JR, Tseng YC, et al. Glycans on influenza hemagglutinin affect receptor binding and immune response. Proc Natl Acad Sci USA. 2009;106(43):18137–18142. doi:10.1073/pnas.0909696106 19822741 PMC2775302

[49] de Vries RP, Smit CH, de Bruin E, et al. Glycan-dependent immunogenicity of recombinant soluble trimeric hemagglutinin. J Virol. 2012;86(21):11735–11744. doi:10.1128/JVI.01084-12 22915811 PMC3486279

[50] Lin SC, Jan JT, Dionne B, et al. Different immunity elicited by recombinant H5N1 hemagglutinin proteins containing pauci-mannose, high-mannose, or complex type N-glycans. PLoS One. 2013;8(6):e66719. doi:10.1371/journal.pone.0066719 23799128 PMC3682957

[51] Zhu S, Nie Z, Che Y, et al. The Chinese hamster ovary cell-based H9 HA subunit avian influenza vaccine provides complete protection against the H9N2 virus challenge in chickens. Viruses. 2024;16(1):163. doi:10.3390/v16010163 38275973 PMC10821000

