# Supplementary material for: Trimeric hemagglutinin vaccine provides chickens complete protection against lethal H5 subtype avian influenza virus from clade 2.3.4.4b
Source: Emerg Microbes Infect. 2026 Jun 10;15(1):2678649. doi: 10.1080/22221751.2026.2678649 (PMC13255209; doi:10.1080/22221751.2026.2678649)
Supplement: Supplementary material.docx [file TEMI_A_2678649_SM7093.docx]

**Supplementary material**

**Supplementary Table 1. Amino acid sequences used in this study**

| >CD33 SP  MPLLLLLPLLWAGALA |
| --- |
| >Trimer-tag GLNGLPGPIGPPGPRGRTGDAGPVGPPGPPGPPGPPGPPSAGFDFSFLPQPPQEKAHDGGRYYRNDDANVVRDRDLEVDTTLKSLSQQIENIRSPEGSRKNPARTCRDLKMCHSDWKSGEYWIDPNQGCNLDAIKVFCNMETGETCVYPTQPSVAQKNWYISKNPKDKRHVWFGESMTDGFQFEYGGQGSDPADVAIQLTFLRLMSTEASQNITYHCKNSVAYMDQQTGNLKKALLLQGSNEIEIRAEGNSRFTYSVTVDGCTSHTGAWGKTVIEYKTTKTSRLPIIDVAPLDVGAPDQEFGFDVGPVCFL |
| >Ferritin (residues 5-167)  DIIKLLNEQVNKEMQSSNLYMSMSSWCYTHSLDGAGLFLFDHAAEEYEHAKKLIIFLNENNVPVQLTSISAPEHKFEGLTQIFQKAYEHEQHISESINNIVDHAIKSKDHATFNFLQWYVAEQHEEEVLFKDILDKIELIGNENHGLYLADQYVKGIAKSRKS |
| >EPM  ALPGNPDHREMGETLPEEVGEYRQPSGGSVPVSPGPPSGLEPTSSSPY |
| >EABR  FNSSINNIHEMEIQLKDALEKNQQWLVYDQQREVYVKGLLAKIFELEKKTETAAHSLP |
| >S836-Trimer  MPLLLLLPLLWAGALADQICIGYHANNSTEQVDTIMEKNVTVTHAQDILEKTHNGKLCRLNGVKPLILKDCSVAGWLLGNPMCDEFISVPEWSYIVERVNPANDLCYPGNLNDYEELKHLLSRINHFEKTRIIPKSSWSSHTSSGVSAACPYQGNASFFRNVVWLTKKNDAYPTIKMSYNNTNKEDLLILWGIHHSNSAEEQTKLYKNPTTYVSVGTSTLNQRLVPKIATRSQVNGQSGRMDFFWTMLKPNDAIHFESNGNFIAPEYAYKIIKTGDSTIMKSEIEYGHCNTKCQTPIGAINSSMPFHNIHPLTIGECPKYVKSNKLVLATGLRNSPLRESSRGLFGAIAGFIEGGWQGMVDGWYGYHHSNEQGSGYAADRESTQKAIDGVTNKVNSIIDIMNTQFEAVGREFNNLERRIENLNKKMEDGFLDVWTYNAELLVLMENERTLDFHDSNVKNLYDKVRLQLRDNAKELGNGCFEFYHKCDNECMESVRNGTYDYPQYSEEARLKREEISGVKLESIGTYQILSIYSTVASSGLNGLPGPIGPPGPRGRTGDAGPVGPPGPPGPPGPPGPPSAGFDFSFLPQPPQEKAHDGGRYYRNDDANVVRDRDLEVDTTLKSLSQQIENIRSPEGSRKNPARTCRDLKMCHSDWKSGEYWIDPNQGCNLDAIKVFCNMETGETCVYPTQPSVAQKNWYISKNPKDKRHVWFGESMTDGFQFEYGGQGSDPADVAIQLTFLRLMSTEASQNITYHCKNSVAYMDQQTGNLKKALLLQGSNEIEIRAEGNSRFTYSVTVDGCTSHTGAWGKTVIEYKTTKTSRLPIIDVAPLDVGAPDQEFGFDVGPVCFLHHHHHH |
| >S836-Ferritin  MPLLLLLPLLWAGALAHHHHHHDQICIGYHANNSTEQVDTIMEKNVTVTHAQDILEKTHNGKLCRLNGVKPLILKDCSVAGWLLGNPMCDEFISVPEWSYIVERVNPANDLCYPGNLNDYEELKHLLSRINHFEKTRIIPKSSWSSHTSSGVSAACPYQGNASFFRNVVWLTKKNDAYPTIKMSYNNTNKEDLLILWGIHHSNSAEEQTKLYKNPTTYVSVGTSTLNQRLVPKIATRSQVNGQSGRMDFFWTMLKPNDAIHFESNGNFIAPEYAYKIIKTGDSTIMKSEIEYGHCNTKCQTPIGAINSSMPFHNIHPLTIGECPKYVKSNKLVLATGLRNSPLRESSRGLFGAIAGFIEGGWQGMVDGWYGYHHSNEQGSGYAADRESTQKAIDGVTNKVNSIIDIMNTQFEAVGREFNNLERRIENLNKKMEDGFLDVWTYNAELLVLMENERTLDFHDSNVKNLYDKVRLQLRDNAKELGNGCFEFYHKCDNECMESVRNGTYDYPQYSEEARLKREEISGVKLESIGTYQILSIYSTVASSSGGDIIKLLNEQVNKEMQSSNLYMSMSSWCYTHSLDGAGLFLFDHAAEEYEHAKKLIIFLNENNVPVQLTSISAPEHKFEGLTQIFQKAYEHEQHISESINNIVDHAIKSKDHATFNFLQWYVAEQHEEEVLFKDILDKIELIGNENHGLYLADQYVKGIAKSRKS |
| >S836-ESCRT  MEKIVLLLSVVDLVKSDQICIGYHANNSTEQVDTIMEKNVTVTHAQDILEKTHNGKLCRLNGVKPLILKDCSVAGWLLGNPMCDEFISVPEWSYIVERVNPANDLCYPGNLNDYEELKHLLSRINHFEKTRIIPKSSWSSHTSSGVSAACPYQGNASFFRNVVWLTKKNDAYPTIKMSYNNTNKEDLLILWGIHHSNSAEEQTKLYKNPTTYVSVGTSTLNQRLVPKIATRSQVNGQSGRMDFFWTMLKPNDAIHFESNGNFIAPEYAYKIIKTGDSTIMKSEIEYGHCNTKCQTPIGAINSSMPFHNIHPLTIGECPKYVKSNKLVLATGLRNSPLRESSRGLFGAIAGFIEGGWQGMVDGWYGYHHSNEQGSGYAADRESTQKAIDGVTNKVNSIIDIMNTQFEAVGREFNNLERRIENLNKKMEDGFLDVWTYNAELLVLMENERTLDFHDSNVKNLYDKVRLQLRDNAKELGNGCFEFYHKCDNECMESVRNGTYDYPQYSEEARLKREEISGVKLESIGTYQILSIYSTVASSLVLAIIVAGLSLWMCSNGSLQCRICIALPGNPDHREMGETLPEEVGEYRQPSGGSVPVSPGPPSGLEPTSSSPYGGGSFNSSINNIHEMEIQLKDALEKNQQWLVYDQQREVYVKGLLAKIFELEKKTETAAHSLPHHHHHH |

**Supplementary Table 2. Program of immunogenicity evaluation test of the candidate HA antigens prepared from HEK293 cells**

| Group | Dose  (per chicken) | Number of animals | Challenge virus |
| --- | --- | --- | --- |
| S836-Trimer | 10 μg in 300 μL | 13 | H5N8-201310-4 |
| S836-Ferritin | 10 μg in 300 μL | 13 |  |
| S836-ESCRT | 10 μg in 300 μL | 13 |  |
| Commercial vaccine | 300 μL | 13 |  |
| PBS | 300 μL | 13 |  |

**Supplementary Table 3. Program of immune efficacy evaluation test of the subunit vaccines based on the antigens from the CHO cells**

| Group | Dose  (per chicken) | Number of animals | Challenge virus |
| --- | --- | --- | --- |
| S836-Trimer (50 μg) | 50 μg in 300 μL | 13 | H5N8-201310-4 |
| S836-Trimer (20 μg) | 20 μg in 300 μL | 13 |  |
| S836-Trimer (10 μg) | 10 μg in 300 μL | 13 |  |
| S836-Trimer Cls (10 μg) | 10 μg in 300 μL | 13 |  |
| Commercial vaccine | 300 μL | 13 |  |
| PBS | 300 μL | 13 |  |

**Supplementary Table 4. Primer sequence used for real-time quantitative PCR.**

| Gene | Primer sequences (5′-3′) | Product size (bp) | Accession no. |
| --- | --- | --- | --- |
| IFN-γ | F: ACCTTCCTGATGGCGTGAAG | 102 | AJ634956.1 |
|  | R: TGAAGAGTTCATTCGCGGCT |  |  |
| IL-4 | F: ATGACATCCAGGGAGAGGTTT | 235 | GU119892.1 |
|  | R: ATTGGAGTAGTGTTGCCTGCT |  |  |
| β-actin | F: TGGGTATGGAGTCCTGTGGT | 136 | NM_205518.1 |
|  | R: CTGTCAGCAATGCCAGGGTA |  |  |

**Supplementary figure captions**

**Figure S1.** **Quantitative analysis of grayscale values of recombinant trimeric HA antigen.** (A) Under the same transfection conditions, the recombinant expression plasmids were transfected into suspension HEK293 cells. Western blot was used to detect the recombinant trimeric HA antigen in the cell culture supernatant or cell lysis supernatant, and protein concentration was quantified based on grayscale values. 1-5: Standard sample 1-5 (Concentrations of 200 μg/mL, 420 μg/mL, 100 μg/mL, 210 μg/mL, and 130 μg/mL, respectively); 6: S836-Trimer; 7: S836-Ferritin; 8: S836-ESCRT; M: 180 kDa protein marker. (B) Standard curve of protein concentration vs. grayscale value.

**Figure S2.** **Histological examination results of organs from experimental chickens.**

**Figure S3.** **The neutralizing antibody titers against the H5N6-J565 strain of the immunized serum.** The serum collected at 10 dpi (A) and 19 dpi (B) was used to measure the cross-neutralizing antibody titers against the H5N6-J565 strain. Statistical significance was indicated by * (*P*<0.05).

**Figure S4. Cytokine secretion level in chicken PBMC and splenocyte in immune efficacy evaluation of the candidate subunit vaccine based on CHO cell-expressed antigens.** PBMCs and splenocytes of chickens from the S836-Trimer (10μg) group, commercial vaccine group, and PBS group were collected at 19 dpi, stimulated with specific purified antigens or inactivated virus, and the mRNA expression levels of cytokine IFN-γ (A, C, E, G) and IL-4 (B, D, F, H) were determined by qRT-PCR. The statistically significant difference was indicated by * (*P*<0.05), ** (*P*<0.01), or *** (*P*<0.001).

**Figure S1**

**
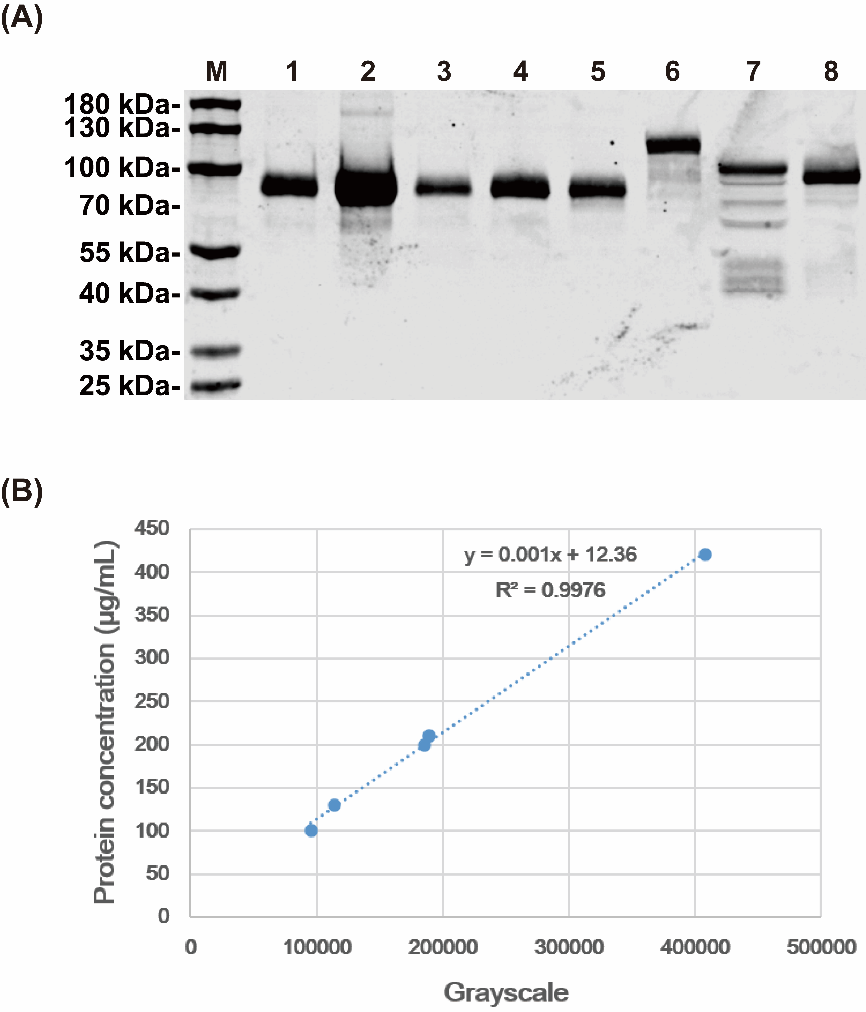
**

**Figure S2**

**
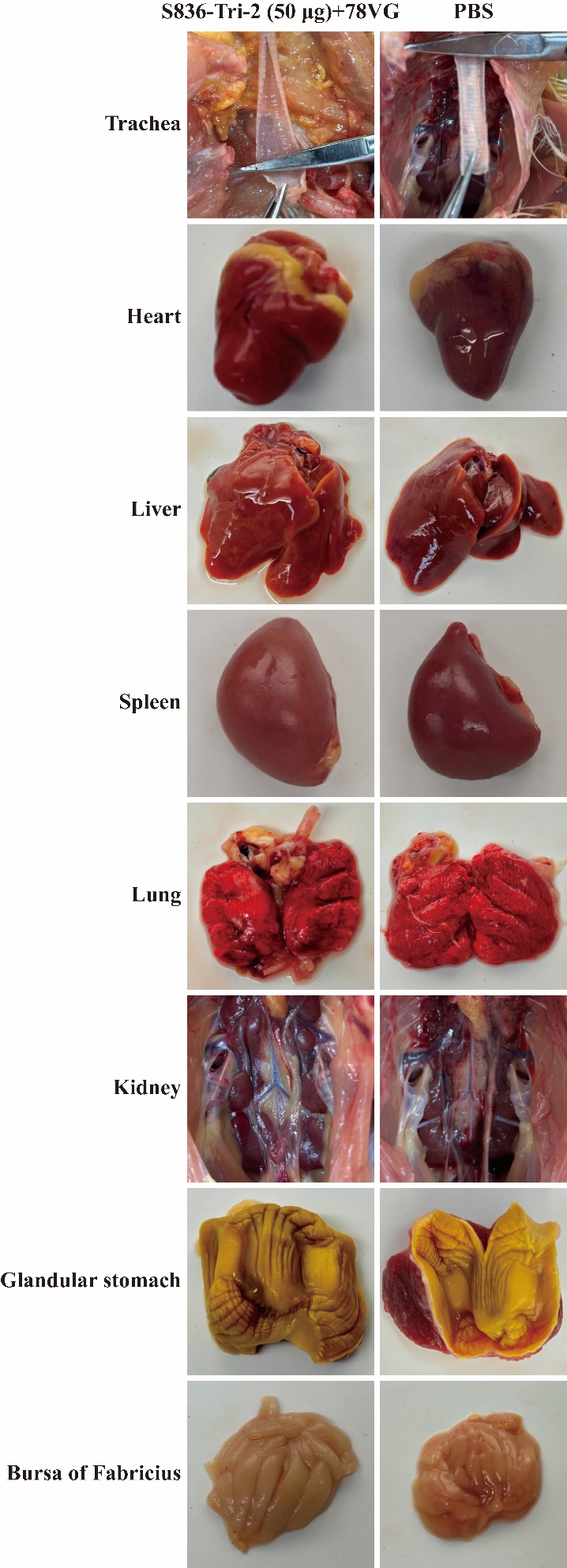
**

**Figure S3**

**
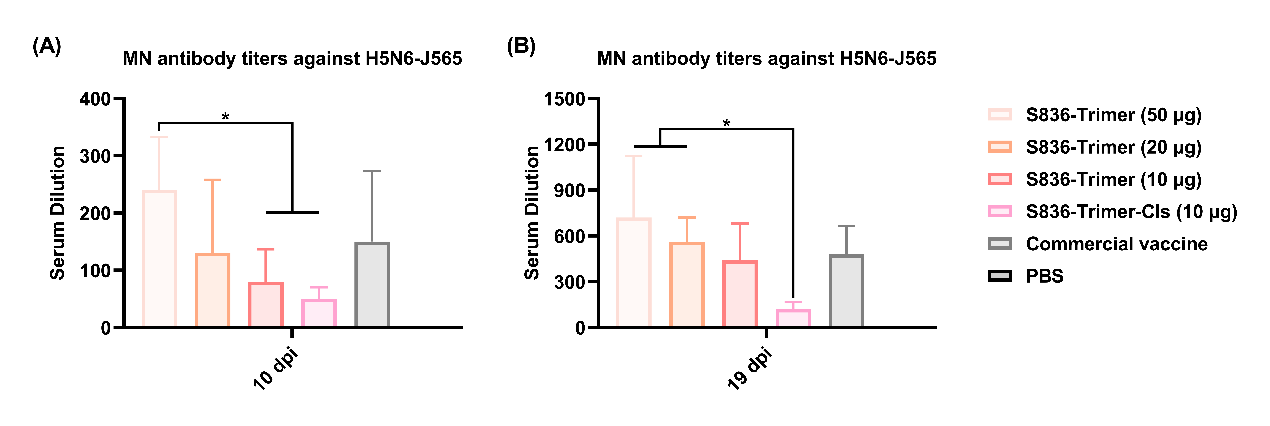
**

**Figure S4**

**
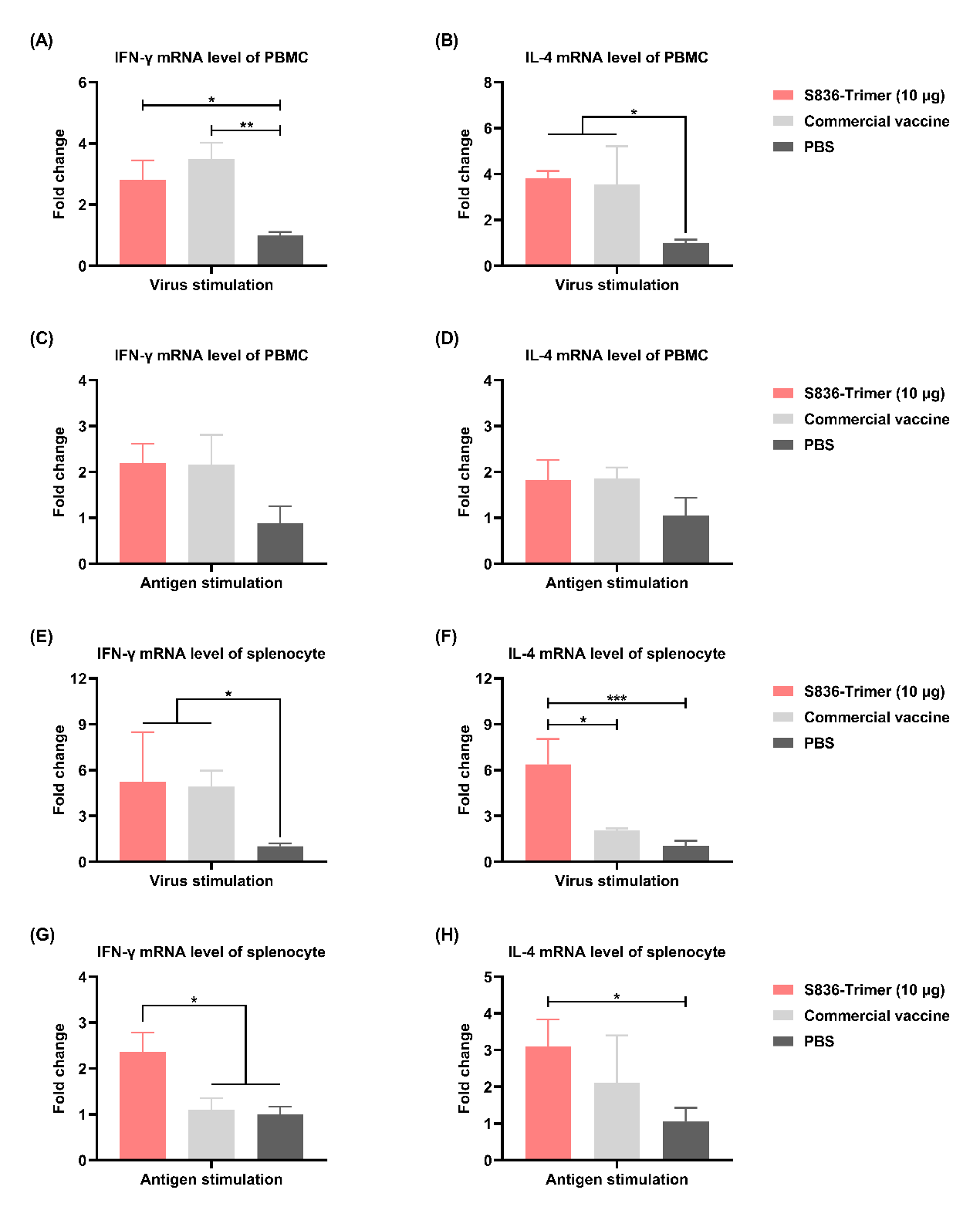
**
